# Honey bee colony loss linked to parasites, pesticides and extreme weather across the United States

**DOI:** 10.1038/s41598-022-24946-4

**Published:** 2022-12-01

**Authors:** Luca Insolia, Roberto Molinari, Stephanie R. Rogers, Geoffrey R. Williams, Francesca Chiaromonte, Martina Calovi

**Affiliations:** 1 Institute of Economics & EMbeDS, Sant’Anna School of Advanced Studies, Pisa, 56127 Italy; 2 Geneva School of Economics and Management, University of Geneva, Geneva, 1205 Switzerland; 3 Department of Mathematics and Statistics, Auburn University, Auburn, 36849 AL USA; 4 Department of Geosciences, Auburn University, Auburn, 36849 AL USA; 5 Department of Entomology and Plant Pathology, Auburn University, Auburn, 36849 AL USA; 6 Department of Statistics, The Pennsylvania State University, University Park, 16802 PA USA; 7 Department of Geography, Norwegian University of Science and Technology, Trondheim, 7491 Norway

**Keywords:** Ecosystem ecology, Environmental impact

## Abstract

Honey bee (*Apis mellifera*) colony loss is a widespread phenomenon with important economic and biological implications, whose drivers are still an open matter of investigation. We contribute to this line of research through a large-scale, multi-variable study combining multiple publicly accessible data sources. Specifically, we analyzed quarterly data covering the contiguous United States for the years 2015-2021, and combined open data on honey bee colony status and stressors, weather data, and land use. The different spatio-temporal resolutions of these data are addressed through an up-scaling approach that generates additional statistical features which capture more complex distributional characteristics and significantly improve modeling performance. Treating this expanded feature set with state-of-the-art feature selection methods, we obtained findings that, nation-wide, are in line with the current knowledge on the aggravating roles of *Varroa destructor* and pesticides in colony loss. Moreover, we found that extreme temperature and precipitation events, even when controlling for other factors, significantly impact colony loss. Overall, our results reveal the complexity of biotic and abiotic factors affecting managed honey bee colonies across the United States.

## Introduction

Honey bees (*Apis mellifera*) are economically important insect pollinators whose widespread loss is increasingly affecting Asia, Europe and North America^1–5^. Between April of 2019 and April of 2020 the United States reported a 43% colony loss^6^. Several factors can contribute to honey bee colony losses, alone or in combination^7,8^. Among the most relevant are parasite and pathogen loads, which in turn depend on beekeepers’ management practices such as the control of *Varroa destructor*^9–15^. Also land use around the colonies^16^, as well as urbanization and agricultural intensiveness^17–21^, play a role by affecting forage quality and pesticide exposure. Relatedly, climate change is considered one of the main drivers of biodiversity loss, in conjunction with agricultural expansion, over-exploitation, and the introduction of invasive species^22^, as it affects the species’ spatial distribution and abundance^23^. Climate^24,25^ and weather changes^26,27^ may consequently play a fundamental role in honey bee colony loss, affecting the availability of forage, thermoregulatory ability during winter, and the initial brood rearing time during spring^28^. Finally, honey bee colony loss varies across time and space, although overwintering survival is generally recognized as the most challenging period of the year^6,29–33^.

To date, some state- or county-level studies investigated the effects of parasites, pathogens, weather, climate change, forage quality and pesticide exposure on honey bee colony loss, often considering one or a few of these factors in isolation and in a controlled environment^17,24,34–37^. In particular, weather factors such as temperature and rainfall were investigated in Switanek et al. (2017) and Beyer et al. (2018)^28,38^, and more recently Calovi et al. (2021)^26^ coupled this information with stressor data, topography, land use, and management factors. To the best of our knowledge, the only study carried out at the level of the United States can be found in Naug (2009)^39^, where honey bee colony loss is analyzed solely as a function of land use information. The insights provided by this body of research still await validation through analyses that employ a broader spatial scale, and consider multiple risk factors simultaneously.

Aside from colony tracking (i.e., migratory operations) and other possible limitations that are typical of large-scale observational studies, one of the main reasons for the lack of analyses at the national level is the absence of same-source or same-resolution databases. The present study aims to fill this gap by making use of several publicly available data sources (see *Data* for details). Because these data differ in spatio-temporal resolution, instead of averaging to the lowest resolution available, we propose a data up-scaling approach that retains some of the information at finer scales through more complex distributional characteristics (see *Data processing*). This up-scaling allows us to model most of the contiguous United States (CONUS) territory using quarterly data from the 2015-2021 (the first quarter being January-March), and thus to rely on state-of-the-art feature selection tools to identify the main statistical predictors of honey bee colony loss. The methodology is complemented with the use of outlier detection techniques that can identify and discard atypical observations, thus limiting their influence on model fitting and effect estimation (see *Statistical model*). Notably, inspection of these atypical observations can itself provide valuable insights on spatio-temporal events with extremely high or low honey bee colony losses.

**Figure 1:**
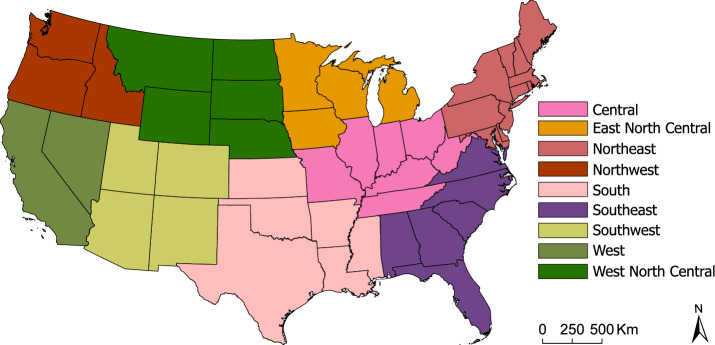
Contiguous United States climatic regions identified by the National Climate Data Center^40^. Climatic regions are presented in different colors for visualization purposes; more detail on the states belonging to each region is provided in Supplementary Fig. S1. The map has been generated by the authors in ArcGIS Pro 2.8.3^41^.

## Results

### Honey bee colony loss and parasites across space and time

**Figure 2:**
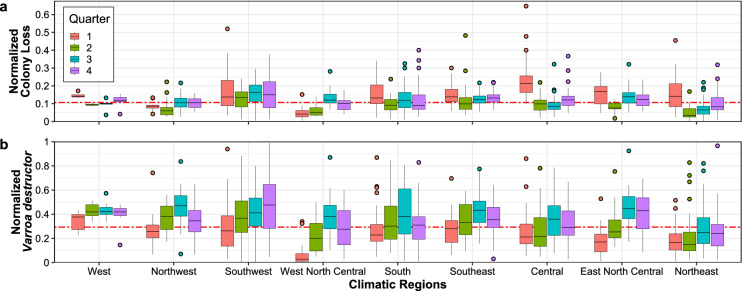
Empirical distribution of honey bee (*Apis mellifera*) colony loss (**a**) and *Varroa destructor* presence (**b**) across quarters (the first one being January-March) and climatic regions; red dashed lines indicate the overall medians. (**a**) Box plots of normalized colony loss (number of lost colonies over the maximum number of colonies) for each quarter of 2015–2021 and each climatic region. At the contiguous United States level, this follows a stable pattern across the years, with higher and more variable losses during the first quarter (see Supplementary Figs. S2-S6), but some regions do depart from this pattern (e.g., West North Central). (**b**) Box plots of normalized *V. destructor* presence (number of colonies affected by *V. destructor* over the maximum number of colonies) for each quarter of 2015–2021 and each climatic region. The maximum number of colonies is defined as the number of colonies at the beginning of a quarter, plus all colonies moved into that region during the same quarter.

**Figure 3:**
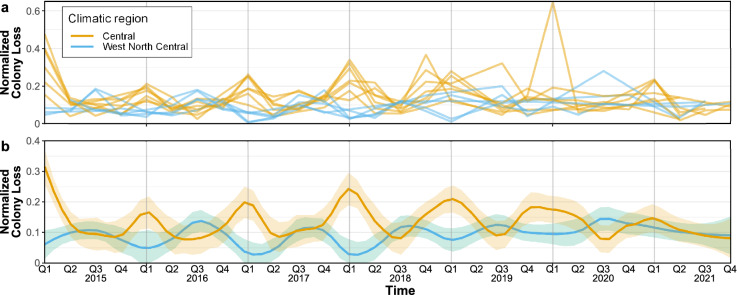
Comparison of normalized honey bee (*Apis mellifera*) colony loss (number of lost colonies over the maximum number of colonies) between Central and West North Central climatic regions for each quarter of 2015–2021 (the first quarter being January-March). (**a**) Trajectory of each state belonging to Central (yellow) and West North Central (blue) climatic regions. (**b**) Smoothed conditional means for each of the two sets of curves based on a locally weighted running line smoother where the width of the sliding window is equal to 0.2 and corresponding standard error bands are based on a 0.95 confidence level^46^.

**Figure 4:**
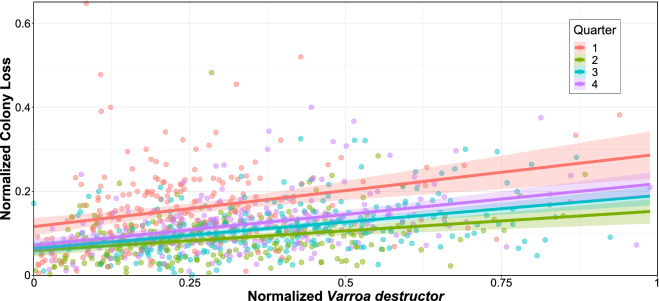
Scatter plot of normalized honey bee (*Apis mellifera*) colony loss (number of lost colonies over the maximum number of colonies) against normalized *Varroa destructor* presence (number of colonies affected by *V. destructor* over the maximum number of colonies) for each state and each quarter of 2015–2021 (the first quarter being January-March). Points are color-coded by quarter, and ordinary least squares fits (with corresponding standard error bands based on a 0.95 confidence level) computed by quarter are superimposed to visualize the positive association.

Honey bee colony loss strongly depends on spatio-temporal factors^33,42^, which in turn have to be jointly modeled with other stressors. Focusing on CONUS climatic regions, defined by the National Centers for Environmental Information^40^ (see Fig. 1), this is supported by the box plots in Fig. 2 which depict appropriately normalized honey bee colony loss (upper panel) and presence of *V. destructor* (lower panel) quarterly between 2015 and 2021. Specifically, Fig. 2a highlights that the first quarter generally accounts for a higher and more variable proportion of losses. Average losses are typically lower and less dispersed during the second quarter, and then tend to increase again during the third and fourth quarters. The Central region, which reports the highest median losses during the first quarter (larger than 20%) exemplifies this pattern, which is in line with existing studies that link overwintering with honey bee colony loss^6,29–33,43^. On the other hand, the West North Central region follows a different pattern, where losses are typically lower during the first quarter and peak during the third. This holds, albeit less markedly, also for Northwest and Southwest regions. These differing patterns are also depicted in Fig. 3, which shows the time series of normalized colony loss for each state belonging to Central and West North Central regions – as well as their smoothed conditional means. Figure 2b shows that also the presence of *V. destructor* tends to follow a specific pattern; in most regions it increases from the first to the third quarter, and then it decreases in the fourth – with the exception of the Southwest region, where it keeps increasing. This is most likely because most beekeepers try to get *V. destructor* levels low by fall, so that colonies are as healthy as possible going into winter, and also because of the population dynamics of *V. destructor* alongside honey bee colonies – i.e., their presence typically increases as the colony grows and has more brood cycles, since this parasite develops inside honey bee brood cells^44,45^. The West region (which encompasses only California since Nevada was missing in the honey bee dataset; see *Data*) reports high levels of *V. destructor* throughout the year, with very small variability. A comparison of Fig. 2a and b shows that honey bee colony loss and the presence of *V. destructor* tend to be higher than the corresponding medians during the third quarter, suggesting a positive association. This is further confirmed in Fig. 4, which shows a scatter plot of normalized colony loss against *V. destructor* presence, documenting a positive association in all quarters. Although with the data at hand we are not able to capture honey bee movement across states, as well as intra-quarter losses and honey production, these preliminary findings can be useful to support commercial beekeeper strategies and require further investigation.

### Up-scaling weather data

The data sets available to us for weather related variables had a much finer spatio-temporal resolution (daily and on a $4 \times 4$ kilometer grid) than the colony loss data (quarterly and at the state level). Therefore, we aggregated the former to match the latter. For similar *data up-scaling* tasks, sums or means are commonly employed to summarize the variables available at finer resolution^47^. The problem with aggregating data in such a manner is that one only preserves information on the “center” of the distributions – thus losing a potentially considerable amount of information. To retain richer weather related information in our study, we considered additional summaries capturing more complex characteristics, e.g., the tails of the distributions or their entropy, to ascertain whether they may help in predicting honey bee colony loss. Within each state and quarter we therefore computed, in addition to means, indexes such as standard deviation, skewness, kurtosis, $L_2$-norm (or energy), entropy and tail indexes^48^. This was done for minimum and maximum temperatures, as well as precipitation data (see *Data processing* for details).

Next, as a first way to validate the proposed weather data up-scaling approach, we performed a likelihood ratio test between nested models. Specifically, we considered a linear regression for colony loss (see *Statistical model*) and compared an ordinary least squares fit comprising all the computed indexes as predictors (the *full model*) against one comprising only means and standard deviations (the *reduced model*). The test showed that the use of additional indexes provides a statistically significant improvement in the fit (*p*-$\text {value}=0.03$). This test, which can be replicated for other choices of models and estimation methods (see Supplementary Table S5), supports the use of our up-scaling approach.

**Figure 5:**
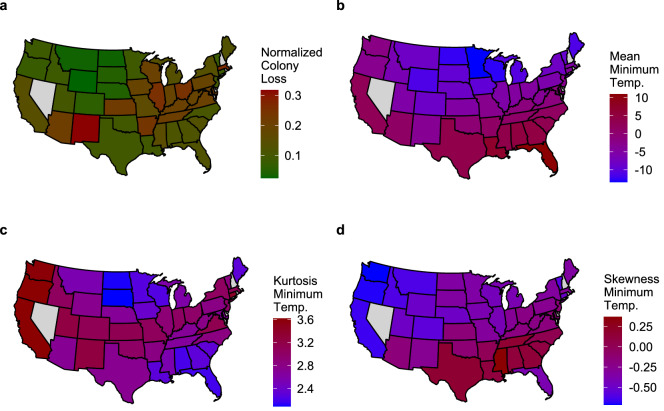
Spatial representation (by state) of median values for four different indices regarding colony loss and minimum temperature in the first quarter (January-March) of seven consecutive years (2015–2021) for each state. (**a**) Normalized honey bee (*Apis mellifera*) colony loss. (**b**) Mean of minimum temperatures. (**c**) Kurtosis of minimum temperatures (how “extreme” the minimum temperatures were). (**d**) Skewness of minimum temperatures (whether they tended to concentrate in their lower or upper range). In each panel, the color attributed to a state represents the median of seven index values (first quarter of seven years). North Dakota shows a relatively low normalized colony loss (panel (**a**)), one of the lowest mean minimum temperature (panel (**b**)), and one of the lowest minimum temperature kurtosis (panel (**c**)). This suggests that consistently low minimum temperatures during the first quarter (low mean and low kurtosis) may be associated with lower colony loss in that state. The map has been generated by the authors in R 3.6.2^49^.

Figure 5 provides a spatial representation of (normalized) honey bee colony losses and of three indexes relative to the minimum temperature distribution; namely, mean, kurtosis and skewness (these all turn out to be relevant predictors based on subsequent analyses; see Table 1). For each of the four quantities, the maps are color-coded by state based on the median of first quarter values over the period 2015-2021 (first quarters typically have the highest losses, but similar patterns can be observed for other quarters; see Supplementary Figs. S12-S14). Notably, the indexes capture characteristics of the within-state distributions of minimum temperatures that do vary geographically. For example, considering minimum temperature, skewness is an index that (broadly speaking) provides information on whether the data tends to accumulate at one end or the other of the observed range of minimum temperatures (i.e., a positive/negative skewness indicates that the data accumulates towards the lower/upper range, respectively). On the other hand, kurtosis is an index that captures the presence of “extreme” values in the tails of the data (i.e., a low/high value of kurtosis indicates that the tail minimum temperatures are relatively close/very far from the typical minimum temperatures). With this in mind, going back to Fig. 5, we can see that minimum temperatures in states in the north-west present large kurtosis (a prevalence of extreme values in the tails) and negative skewness (a tendency to accumulate towards the upper values of the minimum temperature range), while the opposite is true for states in the south-east. More generally, the mean minimum temperature separates northern vs southern states, kurtosis is higher for states located in the central band of the CONUS, and skewness separates western vs eastern states.

We further note that the states with lower losses during the first quarter (e.g., Montana and Wyoming) do not report extreme values in any of the considered indexes. Although these states are generally characterized by low minimum temperatures, these are somewhat “stable” (they do not show marked kurtosis or skewness in their distributions) – perhaps allowing honey bees and beekeepers to adapt to more predictable conditions. On the other hand, states with higher losses during the first quarter such as New Mexico have higher minimum temperatures as well as marked kurtosis, and thus higher chances of extreme minimum temperatures – which may indeed affect honey bee behavior and colony loss. Overall, across all quarters of the years 2015-2021, we found that normalized colony losses and mean minimum temperatures are negatively associated (the Pearson correlation is -0.17 with a *p*-$\text {value}<10^{-6}$ and a sample size of 937). Among all quarters, we also found that the second and the third over the same period showed significantly different kurtosis and skewness of minimum temperatures between states with high and low normalized colony losses (*t*-tests for the difference in mean between minimum temperature kurtosis and skewness for states above and below the overall normalized colony loss median provide *p*-values smaller than $10^{-4}$ and $10^{-3}$, respectively, for a sample size of of 472). Similarly, meaningful associations can be outlined also for other indexes we constructed (see Supplementary Figs. S9-S11), lending additional support to our up-scaling approach. However, these are all “marginal” findings, concerning one potential predictor at a time. Our next task is to move to an analysis that accounts for multiple relationships.

### Joint modeling highlights the roles of *Varroa destructor*, pesticides and extreme weather events

To construct an effective and interpretable model comprising multiple predictors, we need to select which, among the variables at our disposal (including the additional indexes we built by up-scaling weather data), are jointly most predictive of honey bee colony loss. This feature selection exercise is rendered more complex by at least two factors. First, the candidate features we consider, especially the indexes produced by up-scaling, present strong collinearities (see Supplementary Fig. S8). Second, because of the coarse spatio-temporal resolution at which they are measured, most of our variables are likely to aggregate several underlying stochastic mechanisms and thus to contain spurious “contaminations” and outliers that can induce biases and hinder both feature selection and estimation of effects. Hence, while assuming that the majority of the observations are generated by one mechanism (the one being modeled), we need a procedure that can exclude a portion of them from the model fit. For this joint analysis, unlike the descriptive statistics described above, we only consider data covering the years 2015-2019, as honey bee data for 2020-2021 may be affected by the Covid-19 outbreak, and they may also require further validation from the United States Department of Agriculture-National Agricultural Statistics Service (USDA-NASS). Results from an extended analysis covering 2015-2021 are reported in Supplementary Table S13 for comparison, and they are consistent with the ones based on 2015-2019 data.

**Table 1:** Features selected using the mixed-integer programming procedure described in Insolia et al. (2021)^50^ for the years 2015-2019, with corresponding coefficient estimates, standard errors, *t*-statistics and *p*-values computed on a subset encompassing 90% of the observations (these are 607 selected as “non-outlying”, concurrently with the feature selection). Group-constraints are used to ensure that the terms introduced to represent each categorical control, e.g., the three terms representing quarters (the first one being January-March), are either all selected or all excluded (the categorical controls used are year, quarter and climatic region). The model has an $R^2=0.601$.

**Coefficient**	**Estimate**	**Std. Error**	${\varvec{t}}$ **value**	**Pr**${\varvec{(> |t |)}}$
(Intercept)	−2.9903	0.2501	−11.96	$< 2^{-16}$
Year 2015	0.1353	0.0558	2.42	0.0156
Year 2016	0.0231	0.0553	0.42	0.6762
Year 2017	0.0014	0.0541	0.03	0.9788
Year 2018	−0.0202	0.0540	−0.37	0.7080
Region West	−0.3010	0.1050	−2.87	0.0043
Region Northwest	−0.6381	0.0844	−7.56	$<1.6^{-13}$
Region Southwest	−0.0582	0.0939	−0.62	0.5357
Region West North Central	−0.6265	0.0841	−7.45	$<3.5^{-13}$
Region South	−0.1443	0.0634	−2.28	0.0231
Region Southeast	0.0401	0.0562	0.71	0.4755
Region East North Central	−0.1672	0.0618	−2.71	0.0070
Region Northeast	−0.1657	0.0597	−2.77	0.0057
Quarter 1	0.4264	0.0480	8.88	$< 2^{-16}$
Quarter 2	−0.3433	0.0523	−6.57	$< 1.1^{-10}$
Quarter 3	0.0302	0.0744	0.41	0.6845
*Varroa Destructor*	0.1988	0.0209	9.51	$<2^{-16}$
Other pests and parasites	−0.0791	0.0162	−4.88	$<1.4^{-6}$
Pesticides	0.0345	0.0123	2.81	0.0051
Other	0.1524	0.0177	8.60	$< 2^{-16}$
Min. temp. std. dev.	0.0527	0.0205	2.58	0.0102
Min. temp. skewness	0.1777	0.0499	3.56	0.0004
Min. temp. kurtosis	0.5408	0.1187	4.55	$<6.4^{-6}$
Min. temp. alpha index	−0.2357	0.0735	−3.21	0.0014
Max. temp. kurtosis	0.3212	0.1071	3.00	0.0028
Precipitation entropy	0.0875	0.0302	2.90	0.0039
Green-area index	0.1304	0.0352	3.71	0.0002

After transforming normalized colony loss values (per quarter, per state) into log-odds ratios, we regressed them on the features at our disposal. These are 24 features in total, encompassing several stressors (*V. destructor*, pests and parasites, diseases, pesticides, etc.), weather-related information (various indexes computed on minimum and maximum temperatures and precipitation), land use, as well as categorical controls for climatic regions, years and quarters; see *Data* for details. On this set of variables, we applied state-of-the-art statistical learning tools for simultaneous feature selection and outlier detection (see *Statistical model* for details). Specifically, we employed a combinatorial procedure developed by our group^50^, which selects subsets of relevant features and non-outlying points and, on such subsets, is equivalent to an ordinary least squares estimator. Table 1 shows results produced by the procedure when the proportion of outliers to be excluded from the fit is set to 10%. Details on parameter tuning, out-of-sample prediction performance and model diagnostics are provided in Supplementary Figs. S16-S19; similar results obtained with alternative models and estimation methods are discussed in Supplementary Table S8.

Only 15 out of 25 features (including the intercept) were selected as relevant and provided an $R^2=0.6$. In considering estimated coefficients and their signs, recall that negative/positive signs correspond to positive/negative impacts on honey bees (that is, lower/higher colony loss) and that estimates in a joint model need to be interpreted in context (that is, conditional on the other features in the model “being held fixed”). We must note that the following findings take several potential stressors and spatio-temporal controls into account and are thus more complete, informative and interpretable than marginal analyses. Specifically, the descriptive results reported in Figs. 2–5 consider indices in isolation from others and are consequently not fully comparable to the modeling results which take into account several stressors and control variables. All categorical controls were among the relevant features. Signs here are relative to the reference categories, which are “Central” for regions, “2019” for years and “4th” for quarters (these references do not appear in the Table). In terms of spatial effects, Southeast experiences the highest losses; Southwest and Central (the reference category) have similarly high losses, while all other regions show significantly lower losses – with particularly large decreases for Northwest and West North Central (these findings are in line with a separate analysis conducted with state-level controls; see Supplementary Table S9). In terms of temporal effects, years do not appear to have a large impact overall – aside from 2015, when losses were significantly higher. Quarters have a larger impact, with first and second quarters characterized by significantly elevated and reduced losses, respectively – likely due to the fact that most vulnerable colonies die in the Winter, leaving a much healthier population at the start of the Spring (see also the box plots in Fig. 2a; the third quarter estimated coefficient is positive, but the effect is not significant).

Even in conjunction with the spatio-temporal controls, and consistently with existing literature, we found that the presence of *V. destructor*^9–11,15^, the use of pesticides^51–53^ and “other” factors appear to be positively associated with honey bee colony loss^16,28^. Although our findings are not sufficient to draw definite conclusions on causal mechanisms, the statistical associations we documented provide insights and offer hypotheses that can guide future research. Based on the USDA-NASS definition, “other” is a very broad feature which includes factors such as weather, starvation, insufficient forage, queen failure, hive being damaged or destroyed, etc. (see *Data*); we employ it as an additional control variable, which can usefully mitigate confounding effects, but we do not attempt to interpret it – as we do not have a way to assess the role of individual factors within it based on the data at our disposal. Importantly, “other” does provide a significant signal in modeling honey bee colony loss (*p*-$\text {value}<10^{-4}$). Similarly, we notice that the variable “other pests and parasites” (tracheal mites, nosema, hive beetle, wax moths, etc.; see again *Data*) appears to be significant but, as for the variable “other”, we do not attempt to interpret it in depth because of its broad definition – we only use it as a control variable. However, if one were to attempt an interpretation of the negative sign of this variable, it may be, at least in part, due to the presence of collinearities within the selected features; see Supplementary Fig. S19. The marginal correlation between “other pests and parasites” and losses is positive, but this feature is also correlated with *V. destructor*, “other” and “pesticides”, which increase losses^54^. To some extent, these features all capture related effects and disentangling the roles of correlated predictors is non-trivial (see Supplementary Table S7). The negative estimated coefficient when “other pests and parasites” is evaluated jointly with *V. destructor* and “pesticides” could be interpreted as a conditional proxy for the beneficial effect of beekeepers’ expertise, since the pathogens in this category are likely harder to detect and treat compared to, e.g., *V. destructor*. Unfortunately, this hypothesis cannot be validated empirically in the current study, due to the lack of information regarding beekeepers’ expertise in USDA-NASS data. Thus, although the estimated sign for “other pests and parasites” is different than what one would expect based on a marginal analysis, different estimation techniques and modeling strategies support this result (see Supplementary Tables S8-S12). The issue certainly warrants further investigation in the future.

Consistent with our initial likelihood ratio test, also six among the indexes produced by up-scaling weather data were selected as relevant by the procedure. These concern the distributions of minimum temperatures, maximum temperatures and precipitations. Standard deviation, skweness, and kurtosis of minimum temperatures appear to significantly increase losses – suggesting an aggravating role for variability in general, and more specifically for extreme minimum temperature events (concentration on extreme values and tail heaviness of the distribution). This is confirmed by the significant negative effect of the minimum temperatures alpha index – another indicator of the frequency of extreme events; an increase in the index signifies a decrease in extreme events^48^. Extreme maximum temperatures, as captured by their kurtosis, also appear to significantly increase losses, as does the entropy of precipitations. The latter could be interpreted as an effect of the inconsistency of precipitation patterns within a given state and quarter, which may affect the effectiveness of foraging behaviors (bees do not fly during heavy precipitation) and thus increase the probability of colony loss. This supports existing studies connecting colony loss with changing weather patterns^24,26,27,37,43,55,56^.

Finally, the “green-area index”, which captures urbanization^39^ (it is lower/higher for more/less urban areas; see *Data processing* for more detail on the definition), was selected as relevant by our procedure, with a significant positive effect. This suggests that, conditional on all other features included in the model, losses increase when green areas are more abundant. This is in contrast with the result in Naug (2009)^39^ which, however, was based on a regression of losses on land use alone. Indeed, we found that the sign of this relationship does depend on the joint model and data considered, e.g., it can change as one changes the controls included in the model and the set of observations detected as outliers and removed from the fit (see Supplementary Table S8). For instance, using state-level controls in place of regional controls, while not affecting sign and significance of other effects, results in a non-significant negative effect of “green-area index” (*p*-$\text {value}=0.9$; see Supplementary Table S9). The way this index was constructed supports the intuition that it captures state-level variability (see *Data processing*), and its marginal correlation with other selected features is very weak (see Supplementary Figs. S15, S19); further investigation of its association with colony loss conditional on control factors and other features is clearly warranted, also in local-scale studies. We also remark that green areas, particularly crops, may offer transient forage to honey bees, having a detrimental effect on the diversity and availability of forage. Moreover, due to pesticide use, green spaces corresponding to crops may have an additional negative impact on honey bees’ health. To investigate these effects, we decomposed the “green-area index” treating crops and other green areas separately, and found that our results do not change introducing such decomposition. This is likely due to the fact that the two component parts and the overall index are correlated and thus capture similar effects – e.g., the “green-area index” and the index based only on crops have an overall Pearson correlation of 0.84 (this relationship becomes even stronger if we control for states or climatic regions).

In terms of outlier detection, our procedure identified some locations and periods which experienced unexpectedly “high” or “low” honey bee colony losses compared to the overall trend. These terms indicate observations for which the estimated regression residuals are much larger in magnitude than the remaining cases, and are characterized by a positive or negative sign, respectively. Specifically, we found that unexpectedly high losses tend to cluster in the third quarter in West North Central (Nebraska, South Dakota) or Southern regions (Arkansas, Kansas) – i.e., areas where expected losses are low. In contrast, unexpectedly low losses tend to cluster in the third quarter in Northeastern (New Jersey, Vermont) and Southern (Louisiana, Oklahoma) regions. Both types of unexpected events are less frequent in the period with highest expected losses, which is likely due to overwintering impacts, and the year 2015 accounted for a significant number of those (especially with lower losses); see Supplementary Table S6 for details. The distribution of the points detected as outliers deviates from the remaining observations as well. For instance, unexpectedly high losses are associated with lower levels of *V. destructor*, but the opposite holds for unexpectedly low losses. Outlying cases also showed markedly lower levels of the variables “other” and “other pests and parasites” (supporting the fact that they capture additional features of the error distribution compared to the presence of *V. destructor*) and larger values of the “green-area index”; see Supplementary Fig. S20.

## Discussion

Our study explored potential drivers of honey bee colony loss considering the joint effects of a large number of features, controlling for space and time, and covering most of the CONUS territory. Since the open data sources at our disposal were collected at different spatio-temporal resolutions, we introduced an up-scaling approach which allowed us to exploit several distributional characteristics of weather-related variables. This was beneficial in capturing complex relationships and significantly improved the predictive power of our modeling exercise – whose salient findings include key roles for seasonality, location, well-known stressors such as the presence of *V. destructor* and the use of pesticides, as well as weather instability and the prevalence of extreme weather events. Again, we stress that these associations are to be interpreted in light of the limitations of a study that aggregates different data sources – including honey bee surveys that may carry several forms of bias. Hence, our findings are best seen as informative indications – in line with prior studies and motivating future research.

Concerning seasonality, in most states, losses are highest in the first quarter, likely due to overwintering, and lowest in the second quarter, likely due to the beneficial effects of the spring season. This is consistent with existing literature showing that overwintering greatly affects honey bee colony loss^6,26^. Concerning location, keeping all other factors constant, Central, Southeastern and Southwestern regions are generally associated to higher losses throughout the year. Concerning stressors, several local-level studies have provided evidence that *V. destructor* and pesticide use are positively associated to colony losses^57–59^. Our work provides indications that these relations seem to hold also when analyzing broader spatio-temporal scales, and would appear to contradict other local studies that did not observe a positive association with pesticide use^9,60^. However, we remark that pesticide exposure is assessed through survey data that heavily rely on the beekeepers’ knowledge of their colonies. Concerning weather-related variables, e.g., minimum temperatures, we found that measures of variability and tail heaviness (extreme values) significantly increase losses. Interestingly, this is consistent with studies showing that varying temperatures may impact wintering because honey bees go through cycles of clustering^61^. Indeed, one reason for beekeepers to keep honey bees in sheds over the winter, when temperatures are low, is that they can keep them consistent – but this in turn depends on colony metabolic rates and temperature levels^62^. More generally, our findings on the roles of parasites and weather-related variables may inform several aspects of beekeepers’ practices – for instance, how to best move colonies (e.g., to target nectar flows), provide supplementary feed when weather restricts foraging (e.g., via drought), and implement *V. destructor* treatment options depending on weather conditions, including temperatures. Following up on this, while we did not have access to (and could not include in our analyses) information on beekeepers’ practices and colony sizes, these could themselves be among the determinants of honey bee colony loss. Including these information in our modeling exercise would be a very valuable future addition.

From a methodological standpoint, yet more sophisticated statistical approaches could be explored both to up-scale information available at finer resolutions and to construct models. In particular, gains could be made by explicitly accounting for small-scale spatio-temporal dependencies when up-scaling variables, and by using mixed-effects linear models and/or models that comprise broader-scale spatio-temporal dependencies – in addition or as an alternative to the spatio-temporal controls used in the current analyses. Improved modeling strategies may also include the use of second order terms and, in particular, interactions as well as lagged variables – a potential role for these is suggested, for example, by the roughly anti-cyclical behavior over quarters of colony loss and *V. destructor* mites seen in Fig. 2a and b. Although further investigation is needed, some preliminary results (see Supplementary Tables S10–S11) do not appear to point to a significant contribution of these variables in terms of predictive power, and their inclusion leaves the associations presented in Table 1 generally unchanged. For future analyses, it would also be very beneficial to obtain data at finer spatial and temporal resolution, as well as for longer time spans. Finer resolution data for other variables in addition to weather-related ones would allow us, among other things, to further investigate the loss of information due to aggregation, and the effectiveness of up-scaling in limiting such loss. To the best of our knowledge, such data are currently not available for the United States as a whole, but we plan to better study the performance of our up-scaling approach utilizing long, county-level records available for smaller regions.

We note that both the up-scaling approach and the modeling strategy proposed in our work could also be used to study other geographical areas, species and domains – which in turn could be useful for their methodological validation. We are particularly interested in analyzing data concerning crops, where the main interest is in modeling harvest yields which present important differences compared to pollinators (e.g., crops are not generally moved from one place to another).

Our study suggests that, on a large spatio-temporal scale, parasites, extreme weather events and pesticides are among the potential drivers of honey bee colony loss. Finally, we note that our findings could be leveraged to aid beekeepers’ practices, design more focused field experiments, and more generally motivate an increased data collection effort to support our understanding of honey bee colony loss on a large scale. Moreover, through our results on the effects of extreme weather, we provide preliminary insight on the potential effects of climate change, which may be further investigated by extending the spatio-temporal scale of our study. All methods developed and data sets used, as well as metadata and source code to replicate all analyses presented in this work, are publicly available (see Supplementary Data and Source code).

## Methods

In this study we leveraged open data sources concerning three main types of variables; namely (i) honey bee status and stressors, (ii) weather conditions, and (iii) land use (see *Data*). Since the data sets we employed were collected at different spatio-temporal resolutions, we aggregated those at finer resolution quarterly and by state, but we also designed an up-scaling approach based on computing indexes on the distributional characteristics of values within such aggregations (see *Data processing*). To run our analyses, we then employed state-of-the art statistical learning tools for simultaneous feature selection and outlier detection recently developed by our group (see *Statistical model*).

### Data

Honey bee data were obtained from the annual *Honey Bee Colonies Report* released by the USDA-NASS^63^. This summarizes information collected by the USDA-NASS through the Colony Loss Survey. The data are provided on a quarterly basis and by state for the years 2015-2021. They contain information on colony losses, additions and renovations, as well as the presence of specific stressors and signs of illness. Honey bee status and stressor-related variables are listed and described in Supplementary Table S1, and have been used in this study according to their definition as provided by USDA-NASS. We remark that responses submitted to the USDA-NASS questionnaire on honey bee status and stressors depend on the respondents’ knowledge of their colonies. For every given year, quarter and state, our *normalized honey bee colony loss* (whose transformation is the response variable in our modeling exercise) is computed as the number of lost colonies over the maximum number of colonies (colonies at the beginning of the quarter plus all colonies moved into that state during the quarter). We note that only operations that report five or more total colonies are included in the survey, and that beekeepers need to meet criteria on the definition of a farm, such as reporting an agricultural product turnover higher than $1,000 per year. We remark that stressor variables included in our study (e.g., “other” and “other pests and parasites”) have been included based on the official definitions provided by the USDA-NASS reports, which specify the sub-categories that they encompass (see again Supplementary Table S1). Finally, we note that data for Nevada, New Hampshire, Rhode Island and Delaware are not reported, and the second quarter of 2019 is missing due to a recording suspension by the USDA-NASS.

We computed weather-related variables using the Parameter-elevation Regressions on Independent Slopes Model (PRISM)^64^ data covering 2015-2021 at daily resolution, and the whole CONUS area at the resolution of a 4-kilometer-squared grid. For each day and each of the 482,302 grid elements we extracted maximum and minimum temperature, as well as total precipitation (given by the combination of rain and melted snow); see Supplementary Table S2.

Land use information was obtained from the USDA-NASS Cropland Data Layer (CDL)^65^. The raw data are annual (again for the years 2015-2021) and cover the whole CONUS area at the resolution of a 30-meter-squared grid; Supplementary Table S3.

When creating spatial controls, we grouped states based on climate information (Supplementary Fig. S1). Specifically, we relied on the definition of CONUS *climatic regions* provided by the National Centers for Environmental Information^40^. The use of climatic regions (instead of individual states) is an effective way to limit the degrees of freedom devoted to spatial controls in our modeling exercise. The regions take into account historical commonalities in climate conditions and, importantly, also reflect some of the information contained in the weather indexes used in our study.

### Data processing

Given the different spatio-temporal resolutions provided by different open data sources, variables available at finer resolution were aggregated to obtain individual entries with the same (coarser) resolution.

The *weather variables* (temperatures and precipitations) were aggregated across time and space as to obtain entries per quarter per state, and match the resolution of the variables gathered from the *Honey Bee Colonies Report* – including our colony loss response. Ideally, instead of aggregating fine-resolution variables, one could down-scale coarse-resolution ones. However, down-scaling requires the availability of additional information and can undergo different practical challenges making it a non-recommended approach for the data in this study^66,67^. This said, aggregation can induce a considerable loss of relevant information. Given the goals of our study, we thus designed an *up-scaling approach* that partially counters this loss recovering information on the distribution of the values aggregated within quarters and states. In more detail, we expanded the set of features used in our modeling of honey bee colony loss with indexes capturing various aspects of the distributions of weather variables within quarters and states. We of course considered the mean as a measure of *central tendency*, and along with it indexes that capture *spread* (standard deviation), *asymmetries* and *tail-heaviness* (skeweness and kurtosis)^68^. In addition, since events such as anomalous temperatures and precipitations are known to have an important impact on bee survival^69–72^, we computed the tail index (also referred to as tail exponent $\alpha$), which specifically quantifies the *prevalence of extreme values*^73,74^. We also computed the $L_2$-norm or “energy” index, which quantifies the (normalized) overall *magnitude of the signal* comprised in a variable, and the Shannon diversity or “entropy” index, which quantifies the degree of *unpredictability* of the variable, i.e., how close to a uniform is the distribution on its domain^75^.

The *land use data* was employed to compute the *green-area index* whose marginal impact on honey bee colony loss was studied in Naug (2009)^39^. Following the data processing in Naug (2009)^39^, we grouped land-use categories in 6 major classes – “developed”, “forest”, “pasture”, “rangeland”, “crop”, and “water” – computed the area devoted to each class per year per state, and excluded “water” from the analysis. Based on these areas, per year per state, we computed the index as the ratio of green vs urban land; that is, $\frac{{\rm green}}{{\rm urban}} = \frac{({\rm crop} + {\rm forest} + {\rm pasture} + {\rm rangeland})}{{\rm developed}}$. To match the quarterly resolution of the variables gathered from the *Honey Bee Colonies Report*, we simply replicated the yearly green-area index value for each quarter of the same year.

Honey bee stressor variables, which are provided as proportions in the USDA-NASS reports, have been pre-processed using, for all of them, the same statistical approach which is agnostic to the obtained results; see Supplementary Data treatment for details.

### Statistical model

We considered a typical regression model of the form ${\varvec{y}} = {\varvec{X}} {\varvec{\beta }} + {\varvec{\varepsilon }} ,$ where ${\varvec{y}} \in {\mathbb {R}}^{n}$ is the response vector, ${\varvec{\varepsilon }} \in {\mathbb {R}}^{n}$ an error vector which is assumed to follow a Gaussian distribution $N( {\varvec{0}}, \sigma ^2 {\varvec{I}}_n )$, ${\varvec{X}} \in {\mathbb {R}}^{n \times p}$ the design matrix, and ${\varvec{\beta }} \in {\mathbb {R}}^{p}$ the unknown vector of regression coefficients. For the *i*-th state, *j*-th quarter, and *k*-th year, we computed the proportion of lost colonies as $t_{ijk} = \frac{(\text {lost colonies})_{ijk}}{(\text {max colonies})_{ijk}}$ and the corresponding log odds ratio as $y_{ijk} = \log ( \frac{t_{ijk}}{1 - t_{ijk}} ).$ The response vector ${\varvec{y}}$ comprises the scalar terms $y_{ijk}$ stacked into a vector of size $n=880$ (the 44 CONUS states, times a total of 4 quarters in the years 2015-2019). Similarly, the design matrix comprises a column of 880 1’s, for the intercept term, followed by 29 predictor columns, each formed stacking 880 values for states, quarters and years ($p=30$). These include columns for climatic regions, years and quarters (our categorical controls); for each, one category is fixed as reference and all others are encoded through dummies, so that the corresponding regression coefficients represent fixed differential effects relative to the reference (*p* increases to 42 with this parametrization). Also, some of the continuous predictors were transformed to regularize their distributions, and 5 out of the 42 variables were set aside at the outset due to very high correlations with other predictors. Observations with missing values were excluded from the analysis, reducing the sample size to $n=674$; see Supplementary Data treatment for details.

Following an approach developed by our group and described in Insolia et al. (2021)^50^, we considered a trimmed $L_2$-loss function to limit the influence of outlying observations on the fit, and we enforced sparsity in the ${\varvec{\beta }}$ estimates through an additional $L_0$-constraint. The corresponding problem can be formulated as a mixed-integer programming (MIP), where an integer parameter $k_n$ controls the amount of trimming (i.e., the number of largest squared regression residuals which do not affect the fit), and the sparsity level (i.e., the number of non-zero regression coefficient estimates) is controlled through an integer constraint $k_p$. For realistic *n* and *p*, the simultaneous selection of non-outlying units and relevant features is a double combinatorial problem that imposes a huge computational burden. This has been rendered tractable by modern MIP solvers. In general, MIP methods target a global optimum – but also when the algorithm is stopped prior to achieving such optimum, they provide optimality guarantees for their solution. Moreover, the MIP formulation easily allows one to model structured data. We used this to enforce so-called *group sparsity constraints*^76^ for our categorical controls (climatic regions, years and quarters), ensuring that either *all* or *none* of the categories expressing one control variable are retained in the fit. Unlike the outcomes of other existing robust penalization approaches^77^, the MIP solution is equivalent to an ordinary least squares fit computed on the selected subsets of cases and/or features. Notably, under suitable conditions, this approach possesses some desirable statistical properties. In particular, it produces high-breakdown point estimates (i.e., it can tolerate high levels of data contamination), it satisfies the robustly strong oracle property (i.e., it asymptotically behaves as if the true sets of relevant features and non-outlying cases were known in advance), and it is optimal in terms of prediction errors^50^. Thus, although finite-sample inference can be problematic with this class of techniques as it depends on the selection process, the inferential results in Table 1 can be interpreted in terms of large-sample theory. In Supplementary Fig. S21 and Table S8 we present results obtained using alternative estimation methods and models, which are all consistent with the results presented in the main text.

## Supplementary Information

Supplementary Information 1.

Supplementary Information 2.

## Data Availability

The raw data that support the findings in this paper are openly available at https://usda.library.cornell.edu/concern/publications/rn301137d (United States Department of Agriculture), https://nass.usda.gov/Research_and_Science/Cropland/Release/index.php (United States Department of Agriculture Cropland Data Layer), and https://www.prism.oregonstate.edu/ (Parameter-elevation Regressions on Independent Slopes Model Climate Group). Our source code to process and combine these data sources is available at: https://github.com/LucaIns/honey_bee_loss_US_scirep, and the resulting combined dataset is part of the Supplementary Information.
