# Publisher Correction: Honey bee colony loss linked to parasites, pesticides and extreme weather across the United States

**DOI:** 10.1038/s41598-023-28374-w

**Published:** 2023-01-23

**Authors:** Luca Insolia, Roberto Molinari, Stephanie R. Rogers, Geoffrey R. Williams, Francesca Chiaromonte, Martina Calovi

**Affiliations:** 1 Institute of Economics & EMbeDS, Sant’Anna School of Advanced Studies, Pisa, 56127 Italy; 2 Geneva School of Economics and Management, University of Geneva, Geneva, 1205 Switzerland; 3 Department of Mathematics and Statistics, Auburn University, Auburn, 36849 AL USA; 4 Department of Geosciences, Auburn University, Auburn, 36849 AL USA; 5 Department of Entomology and Plant Pathology, Auburn University, Auburn, 36849 AL USA; 6 Department of Statistics, The Pennsylvania State University, University Park, 16802 PA USA; 7 Department of Geography, Norwegian University of Science and Technology, Trondheim, 7491 Norway

Correction to: *Scientific Reports* https://doi.org/10.1038/s41598-022-24946-4, published online 01 December 2022

The Data availability section in the original version of this Article was incomplete.

“The raw data that support the findings in this paper are openly available at https://usda.library.cornell.edu/concern/publications/rn301137d (United States Department of Agriculture), https://nass.usda.gov/Research_and_Science/Cropland/Release/index.php (United States Department of Agriculture Cropland Data Layer), and https://prism.oregonstate.edu (Parameter-elevation Regressions on Independent Slopes Model Climate Group). Our source code to process and combine these data sources, as well as the resulting combined dataset itself, is part of the Supplementary Information.”

now reads:

“The raw data that support the findings in this paper are openly available at https://usda.library.cornell.edu/concern/publications/rn301137d (United States Department of Agriculture), https://nass.usda.gov/Research_and_Science/Cropland/Release/index.php (United States Department of Agriculture Cropland Data Layer), and https://www.prism.oregonstate.edu/ (Parameter-elevation Regressions on Independent Slopes Model Climate Group). Our source code to process and combine these data sources is available at: https://github.com/LucaIns/honey_bee_loss_US_scirep, and the resulting combined dataset is part of the Supplementary Information.”

The original Article has been corrected.

