# Supplementary material for: Honey bee colony loss linked to parasites, pesticides and extreme weather across the United States
Source: Sci Rep. 2022 Dec 1;12:20787. doi: 10.1038/s41598-022-24946-4 (PMC9714769; doi:10.1038/s41598-022-24946-4)
Supplement: Supplementary file 1 — Supplementary Information 1. [file 41598_2022_24946_MOESM1_ESM.pdf]

Supplementary Information for "Honey bee colony loss linked to parasites, pesticides and extreme weather across the United States"

Luca Insolia[1,2], Roberto Molinari[3], Stephanie R. Rogers[4], Geoffrey R. Williams[5], Francesca Chiaromonte[1,6] and Martina Calovi[7*]

[1]Institute of Economics & EMbeDS, Sant'Anna School of Advanced Studies, Pisa, 56127, Italy.
[2]Geneva School of Economics and Management, University of Geneva, Geneva, 1205, Switzerland.
[3]Department of Mathematics and Statistics, Auburn University, Auburn, 36849, AL, USA.
[4]Department of Geosciences, Auburn University, Auburn, 36849, AL, USA.
[5]Department of Entomology and Plant Pathology, Auburn University, Auburn, 36849, AL, USA.
[6]Department of Statistics, The Pennsylvania State University, University Park, 16802, PA, USA.
[7]Department of Geography, Norwegian University of Science and Technology, Trondheim, 7491, Norway.

*Corresponding author(s). E-mail(s): martina.calovi@ntnu.no; Contributing authors: luca.insolia@santannapisa.it; robmolinari@auburn.edu; s.rogers@auburn.edu; grw0010@auburn.edu; fxc11@psu.edu;

Data treatment

For our linear modeling exercise, after the data processing phase (see the *Data processing* Section of the main text), we further manipulated the data as follows. Honey bee stressor variables such as "*Varroa destructor*", "other pests and parasites", "diseases", "pesticides", and "other" were modeled taking the logit transformation of the proportion of colonies affected by these stressors (similarly to what we did for the response variable; see *Statistical model* Section of the main text). Regarding honey bee status and stressor data, uninformative predictors for honey bee colony loss were excluded from the analysis, namely: "number of initial colonies", "percentage of lost colonies", "percentage of renovated colonies", "unknown", "number of added colonies", and "number of renovated colonies" (see Table S1). For the weather indexes that we built (see Table S2), "alpha indexes", and "kurtosis" of weather variables and the "green-area index" (see Table S3) were log-transformed to mitigate the skewness of their distributions (see Fig. S7). The "precipitation alpha index" was not considered in our analysis due to its extremely concentrated distribution, and we divided the "norms" of weather indexes by a factor of 10^4 to limit their scale. Observations with any missing entry were excluded from the analysis, bringing the sample size from $n = 880$ to $n = 674$. Furthermore, due to strong correlations in the collection of weather indexes that we built in our up-scaling procedure (see Fig. S8), some features were removed at the outset. Specifically, if the absolute pairwise Pearson correlation between two predictors exceeded the cut-off of 0.9, then the variable with higher mean absolute correlation (with respect to all the remaining features) was excluded from the model. This was performed through the `findCorrelation()` function of the `caret R` package (Kuhn, 2009). The predictors excluded in this way include: "minimum temperature mean", "maximum temperature entropy", "maximum temperature alpha index", "precipitation mean", and "precipitation kurtosis". See Table S4 for a description of the predictors used in the linear modeling exercise described in the main text.

Robust feature selection parameter tuning

We used mixed-integer programming (MIP) techniques for simultaneous feature selection and outlier detection developed by our group (Insolia et al., 2021) to obtain the regression results presented in Table 1 of the main text (see also the *Statistical model* Section of the main text). Operationally, one has to estimate a suitable level of trimming k_n, which represents the number of points not affecting the fit, as well as a sparsity parameter k_p, controlling the number of estimated regression coefficients which are non-zero. We explored several combinations of k_n and k_p values; in the following we present the results for k_n/n ranging from 0 to 15% (with a step size of 5%), and k_p ranging from 1 to 30 (with a step size of 1 and counting each dummy variable separately although we use group constraints). For each combination of k_n and k_p values, we let the MIP algorithm run for 5,000 seconds or stop at a 1% optimality gap.

To measure the overall goodness of fit, we computed the robust information criterion discussed in Insolia et al. (2021) with an additional correction factor based on the truncated normal as in Riani et al. (2022). Figure S16 shows the *robust Akaike information criterion* (rAIC) path across different trimming levels, where we report k_n/n on the x-axis. For each trimming level, we estimate the best model of size $1 \leq \widehat{k}_p \leq 30$, and then select the one with minimum rAIC. Here, we see that lower levels of trimming favor sparser solutions, which is likely due to the effects of outlying cases, and that there is little difference in terms of minimum rAIC for trimming levels equal to 5% or 10%. For this reason, instead of picking the solution associated with the overall minimum rAIC, we also compared predictive performance to guide our choice for the trimming level. Specifically, we randomly split the data and used 80% of the observations as a training set and the remaining 20% as a testing set. For each combination of k_n and k_p values, this procedure was repeated 8 independent times, and we computed the *trimmed root mean square prediction error* (TRMSPE) on testing data across models selected by rAIC (with upper trimming equal to the trimming level in use). The left panel of Figure S17 compares the medians plus/minus median absolute deviations (MAD) of the TRMSPE across the 8 random splits for MIP against the ones obtained by the sparseLTS (Alfons et al., 2013) – the latter is an heuristic algorithm for robust feature selection and is computed through the `sparseLTS()` function of the `robustHD` `R` package (Alfons, 2021). The TRMSPE is typically non-increasing, and our MIP procedure outperforms sparseLTS as the trimming level reaches 10%. Moreover, the TRMSPE for MIP has a much smaller variability (which is measured by the MAD) as the contamination level reaches 10%, indicating that this solution is more stable than others. We also took the sparsity of the MIP and sparseLTS solutions into account when comparing predictive performance. The right panel of Figure S17 compares medians and MADs of the selected models of size $\widehat{k}_p$, for each trimming level, across the 8 random splits. For a 10% trimming, MIP not only performs better than sparseLTS in terms of predictive power, but also provides sparser and thus more interpretable solutions (lower median and narrower MAD). Here the median sparsity level for MIP is close to the one that we have found on the full data set (see Figure S16), and it is much more stable than the solutions with a 5% trimming – whose MAD is approximately twice as large.

In light of all these findings, we chose to present the results for a 10% trimming, and we verified that different trimming levels (say, from 5 up to 15%) provide results which are consistent with the ones discussed in the main text. Furthermore, Figure S18 contains several regression diagnostics supporting the fact that the model selected by rAIC with a 10% trimming satisfies the underlying assumptions (for the set of non-outlying cases), and highlights the presence of outlying cases which deserve further investigation (e.g., the presence of some residuals which are 6 standard deviations away from the robust fit). Figure S20 and Table S6 provide further analyses on the outlying cases detected by our MIP.

Computations for this research were performed on the Roar supercomputer of the Institute for Computational and Data Sciences at the Pennsylvania State University. The content of the research is solely the responsibility of the authors and does not necessarily represent the views of the Institute for Computational and Data Sciences. We used basic memory option on the ACI-B cluster with an Intel Xeon 24 core processor at 2.2 GHz and 128 GB of RAM. The multi-thread option in `R` and `Gurobi` was limited to a maximum of 24 threads.

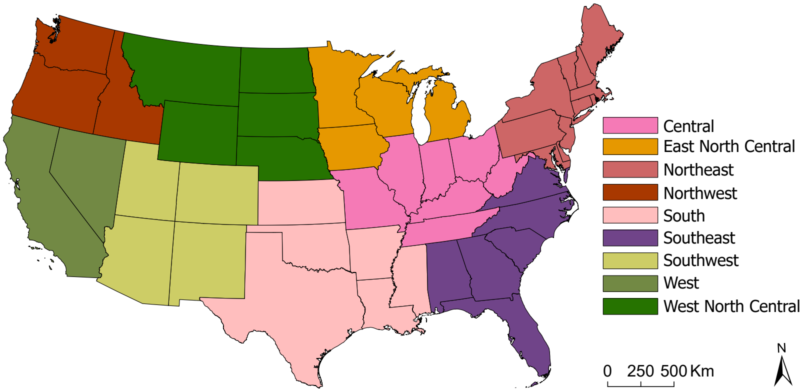

Fig. S1: Contiguous United States climatic regions identified by the National Climate Data Center (Karl and Koss, 1984). They are aggregated as follows. *West region*: California and Nevada; *Northwest region*: Washington, Oregon and Idaho; *Southwest region*: Utah, Colorado, Arizona and New Mexico; *West North Central region*: Montana, Wyoming, North and South Dakota, and Nebraska; *South region*: Kansas, Oklahoma, Texas, Arkansas, Louisiana and Mississippi; *Southeast region*: Alabama, Florida, Georgia, North and South Carolina, and Virginia; *Central regional*: Missouri, Illinois, Indiana, Kentucky, Tennessee, Ohio and West Virginia; *East North Central region*: Iowa, Minnesota, Wisconsin and Michigan; *Northeast region*: Pennsylvania, Washington D.C., Maryland, Delaware, New Jersey, Connecticut, Rhode Island, Massachusetts, New Hampshire, Vermont and Maine. The map has been generated by the authors in ArcGIS Pro 2.8.3 Redlands (2021).

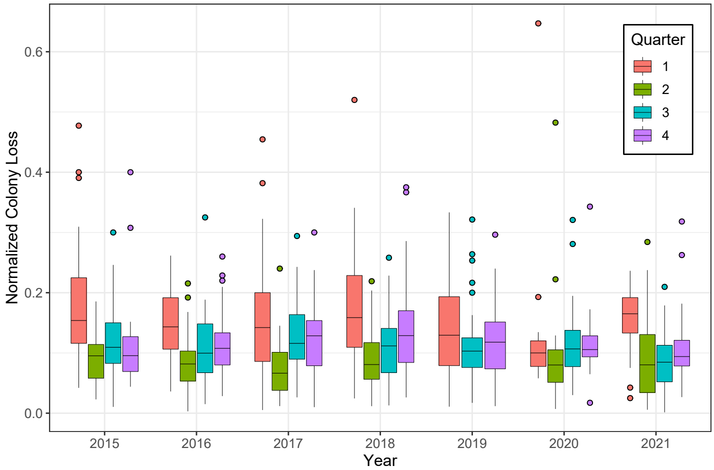

Fig. S2: Box plots of normalized colony loss (number of lost colonies over the maximum number of colonies) for each quarter between 2015 and 2021 across the United States; the second quarter of 2019 was not reported by the United States Department of Agriculture. The figure highlights a stable pattern across the years, showing that the first quarter typically accounts for a sensibly higher proportion of losses and has a larger variability. The second quarter of each year (which is missing for 2019) generally accounts for lowest levels of losses and reports a lower variability compared to other quarters. Losses tend to increase again during the third and fourth quarters. Only in 2015 median losses across the third quarter are higher than the ones during the fourth quarter, but they have larger variability.

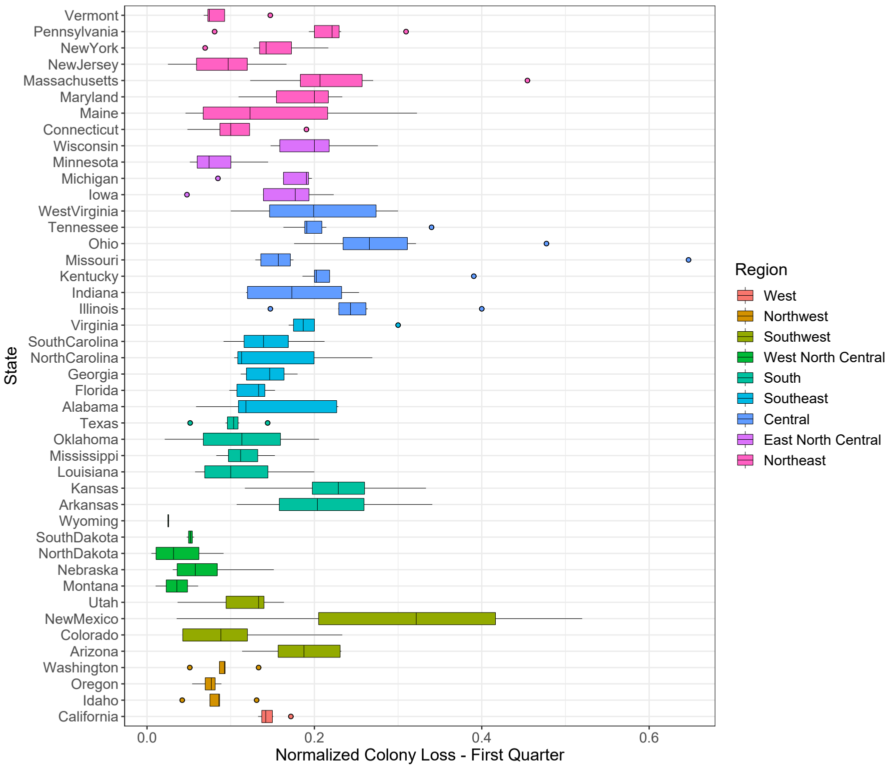

Fig. S3: Box plots of the normalized colony loss (number of lost colonies over the maximum number of colonies) in the first quarter of the years 2015-2021 for different states of the United States aggregated according to climatic regions. It is possible to distinguish a pattern, where states belonging to the same climatic region tend to behave quite similarly. The West North Central and Northwest regions report sensibly lower losses (whose medians are smaller than 10%) characterized by a much smaller variability. On the other hand, many states in the Central region, as well as New Mexico (which has the highest median and variability), Arkansas, Kansas, Pennsylvania, Massachusetts, Ohio and Illinois, report a median loss which is higher than 20%.

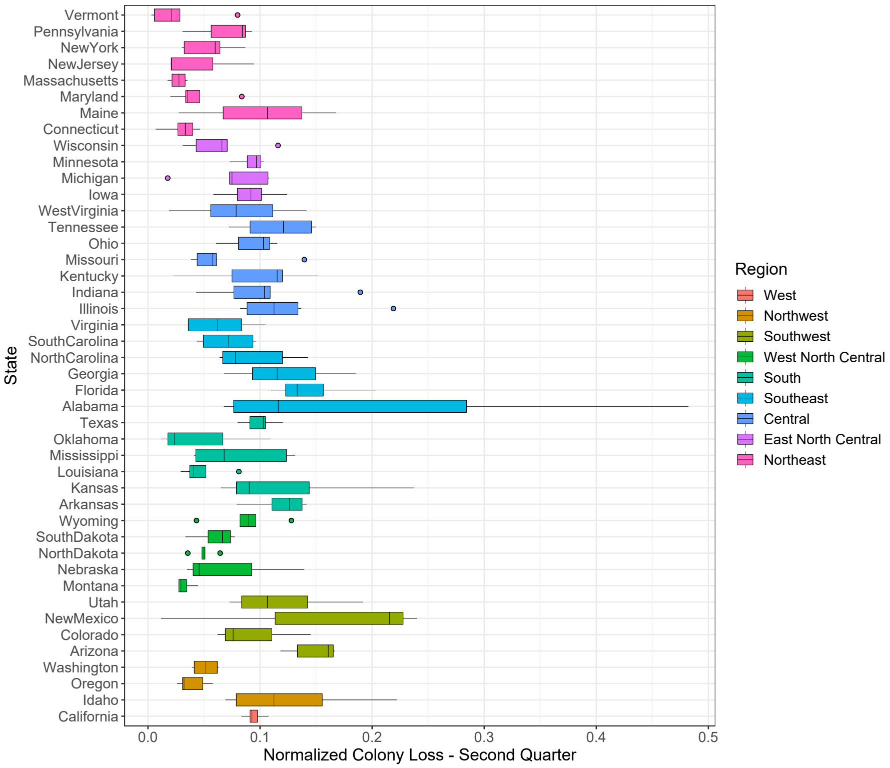

Fig. S4: Box plots of the normalized colony loss (number of lost colonies over the maximum number of colonies) in the second quarter of the years 2015-2021 (2019 data were not reported by the United States Department of Agriculture) for different states of the United States aggregated according to climatic regions. The findings are similar to the ones discussed in Fig. S3, although colony loss is generally lower and the differences are less marked. Alabama reports high extreme losses, whose highest level is achieved during 2020 and is also associated to the largest number of added and renovated colonies (results not shown).

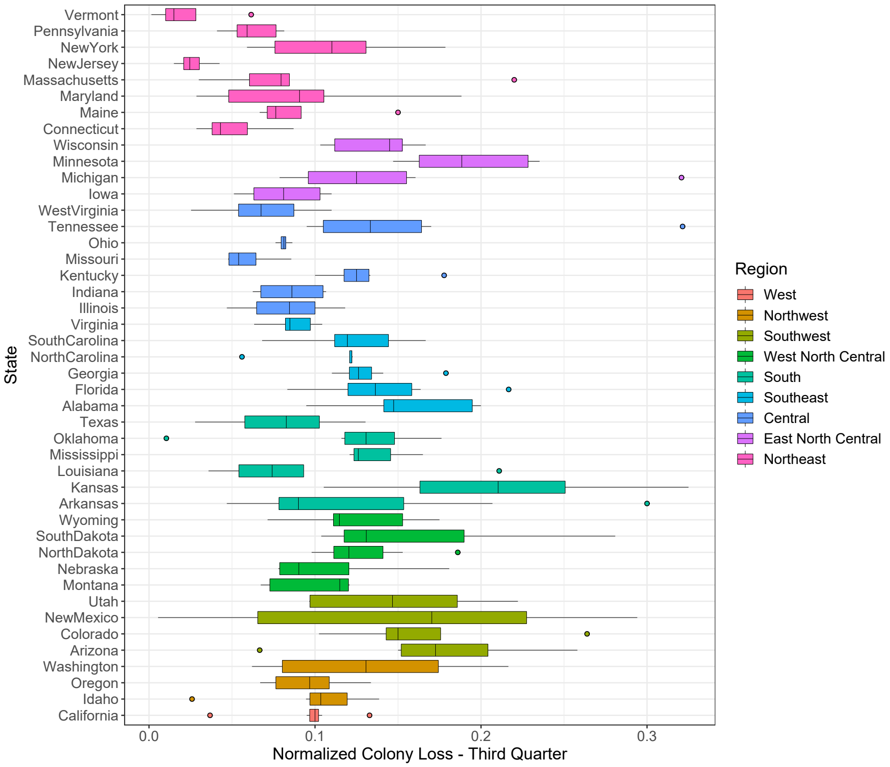

Fig. S5: Box plots of the normalized colony loss (number of lost colonies over the maximum number of colonies) in the third quarter of the years 2015-2021 for different states of the United States aggregated according to climatic regions. Similar findings to the ones discussed in Fig. S3 hold also here, although less markedly. West North Central and Northwest regions report higher losses compared the first quarter (whose medians are typically higher than 10%), and most states in Central region report their lowest levels of losses among all quarters.

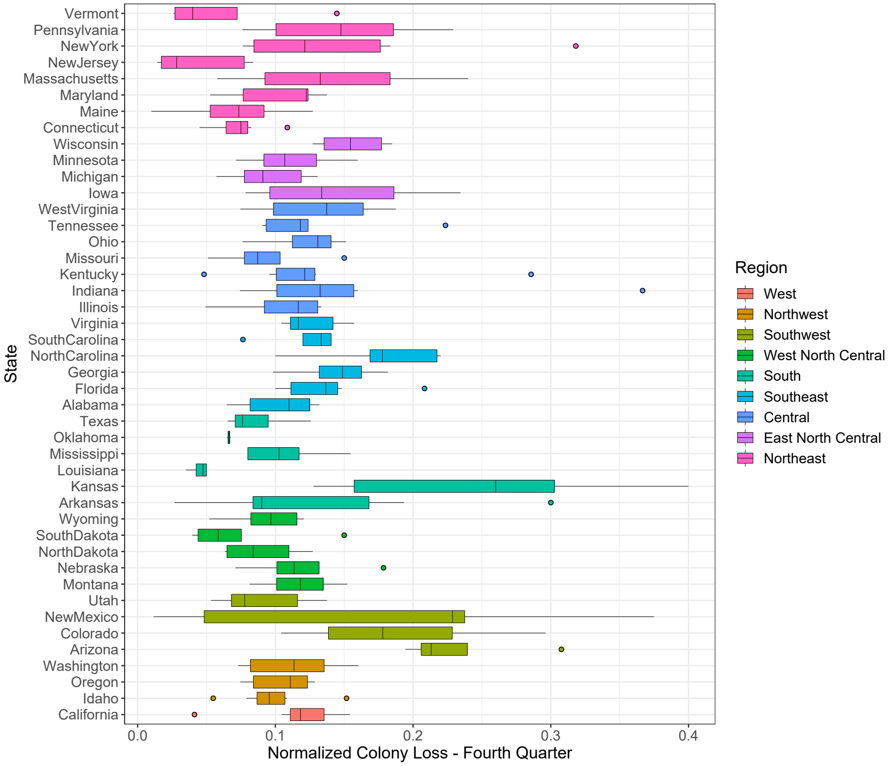

Fig. S6: Box plots of the normalized colony loss (number of lost colonies over the maximum number of colonies) in the fourth quarter of the years 2015-2021 for different states of the United States aggregated according to climatic regions. The findings are similar to the ones discussed in Fig. S3, although also here honey bee loss is generally lower for most states and the differences are less marked. Kansas reports high levels of losses, which are sensibly higher than other states in the South region and somehow more stable compared to the ones of New Mexico. The latter has a long left tail and reports median losses which are comparable to most states in the Southwest region.

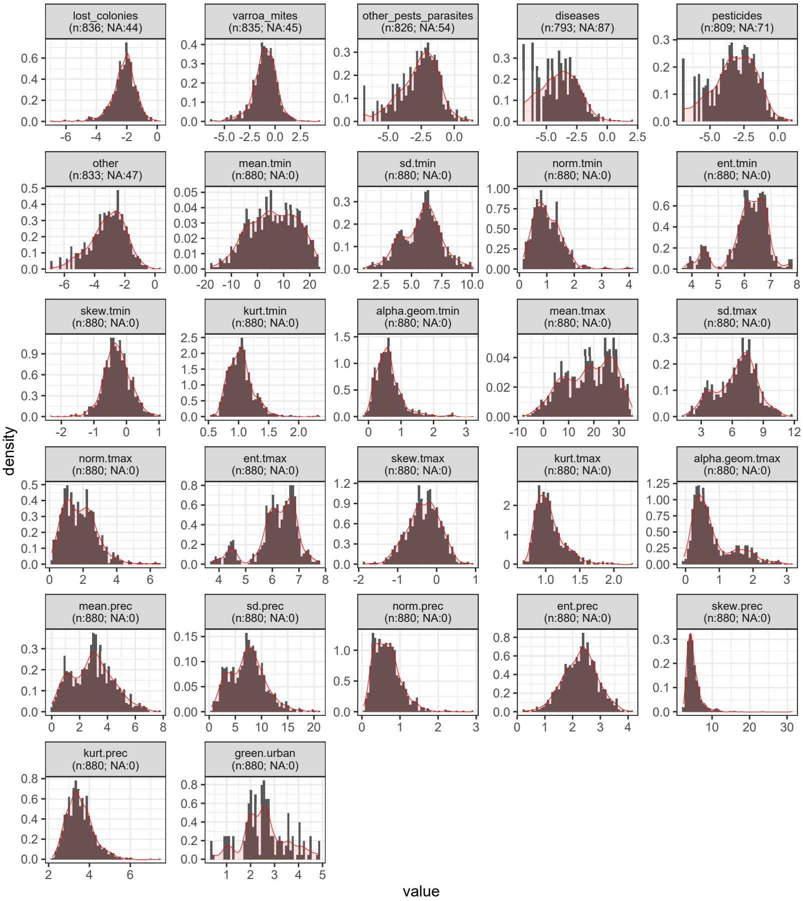

Fig. S7: Distribution of the response variable (lost colonies) and continuous predictors included into our linear modeling exercise for the years 2015-2019 after their transformation (before the removal of collinear predictors). Kernel density estimates are superimposed to histograms (red lines), and the number of points (n) as well as the number of missing data ("NA") are provided for each feature.

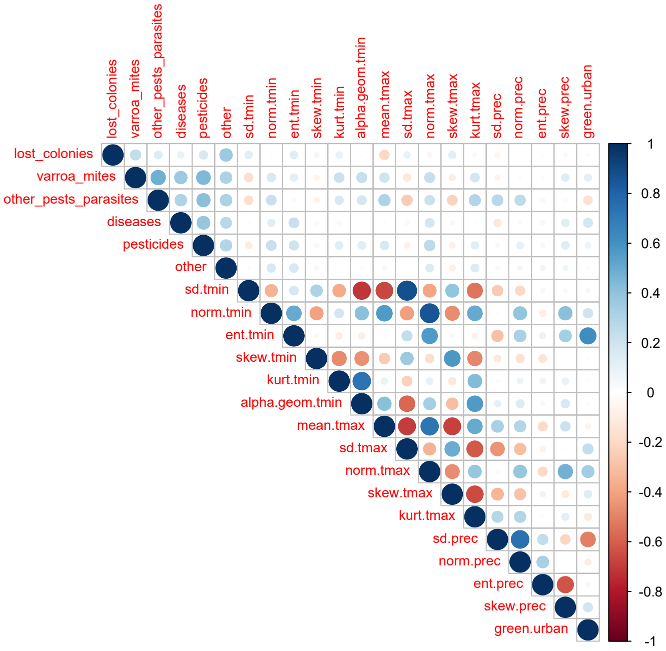

Fig. S8: Pearson correlations for the response variable (lost colonies) and continuous predictors considered in our linear modeling exercise for the years 2015-2019 (after the removal of predictors with Pearson correlations higher than 0.9 as described in the *Data treatment* Section of the Supplementary Information). Unlike stressor variables, which do not correlate much one with another, several weather indexes – that we created to capture more complex distributional characteristics – tend to create groups of correlated features.

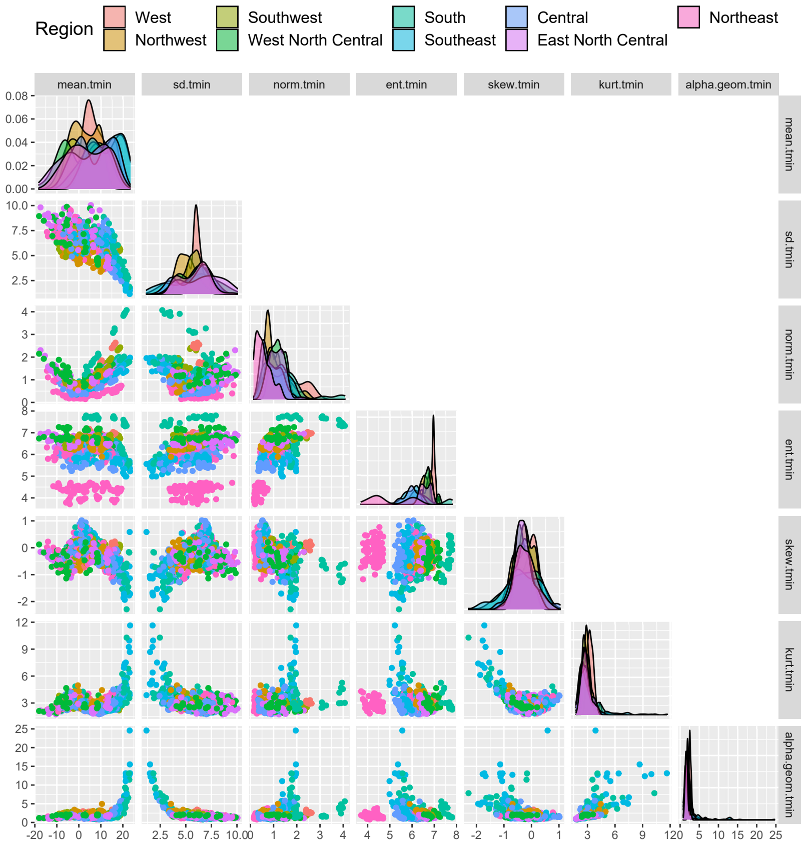

Fig. S9: Scatter matrix and univariate densities for weather indexes created from the distribution of minimum temperatures across the United States climatic regions. These data cover the years 2015-2021.

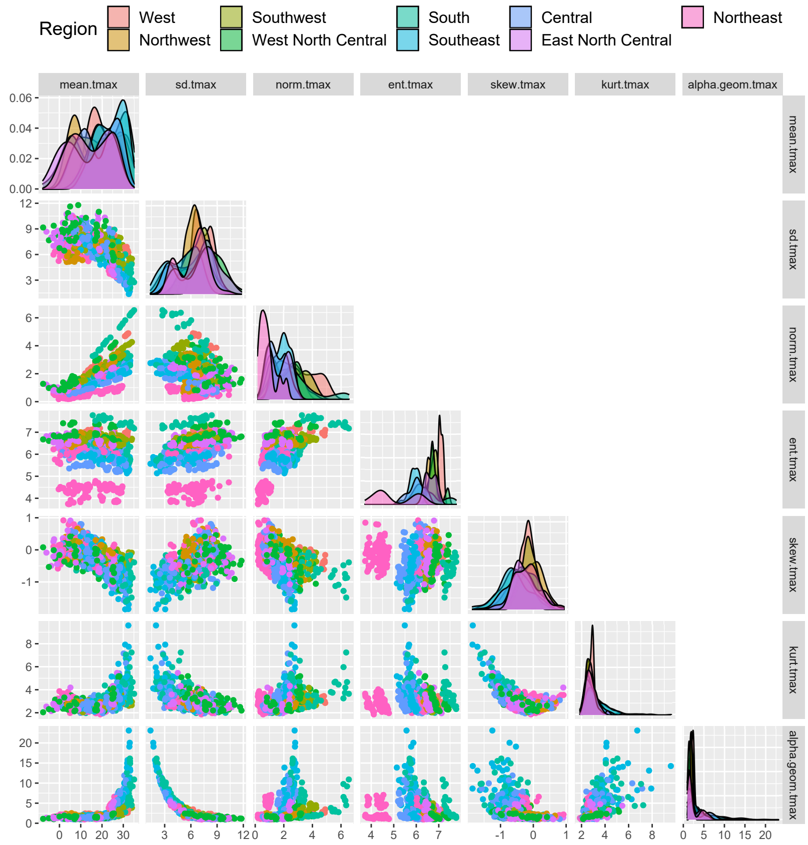

Fig. S10: Scatter matrix and univariate densities for weather indexes created from the distribution of maximum temperatures across the United States climatic regions. These data cover the years 2015-2021.

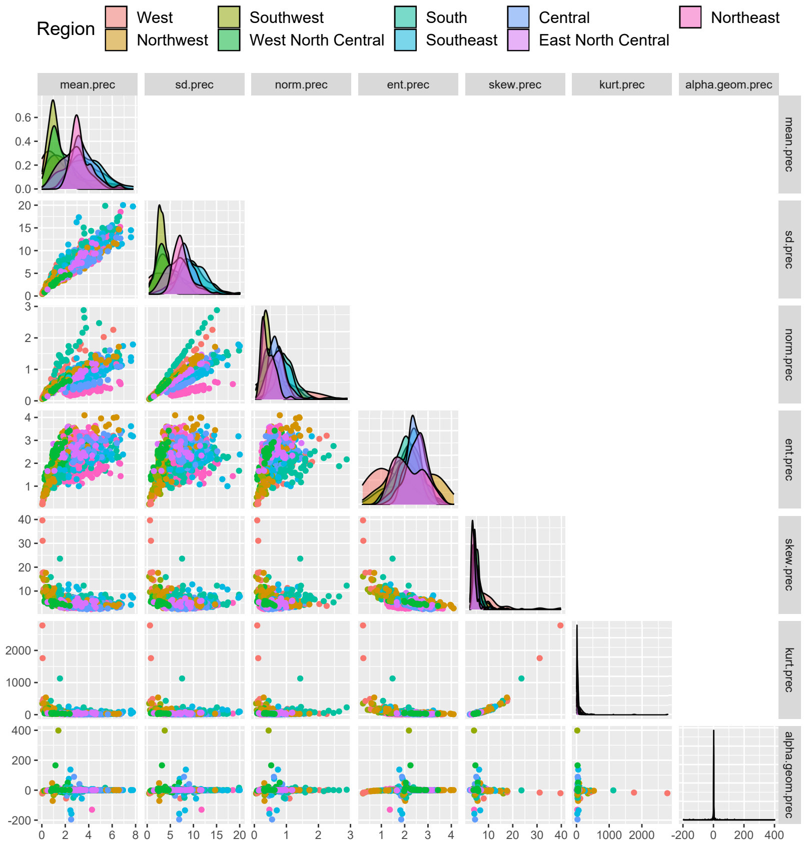

Fig. S11: Scatter matrix and univariate densities for weather indexes created from the distribution of precipitations across the United States climatic regions. These data cover the years 2015-2021.

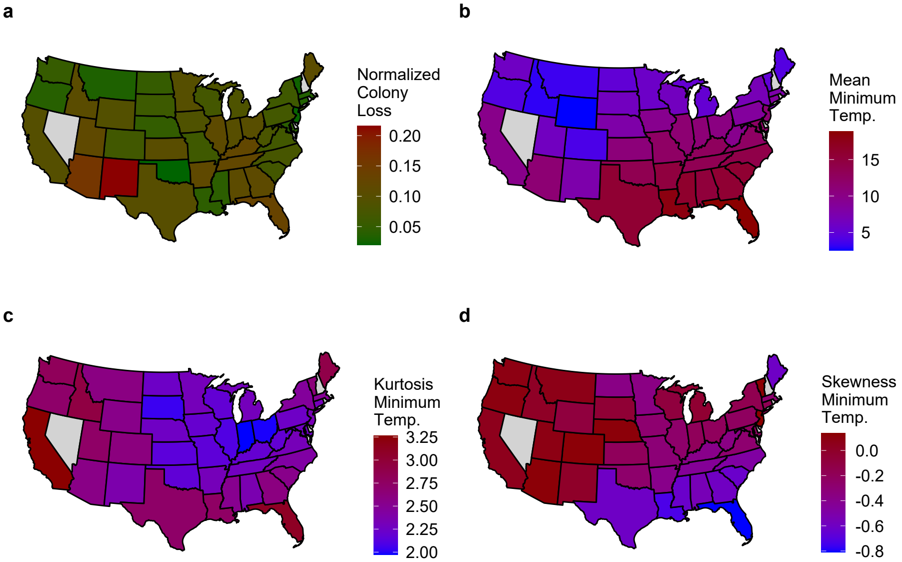

Fig. S12: Spatial representation of the median for second quarter data in the years 2015-2021 (2019 was not reported by the United States Department of Agriculture). (a) Normalized colony loss. (b) Mean of minimum temperatures. (c) Kurtosis of minimum temperatures. (d) Skewness of minimum temperatures. The map has been generated by the authors in R 3.6.2 R Core Team (2021).

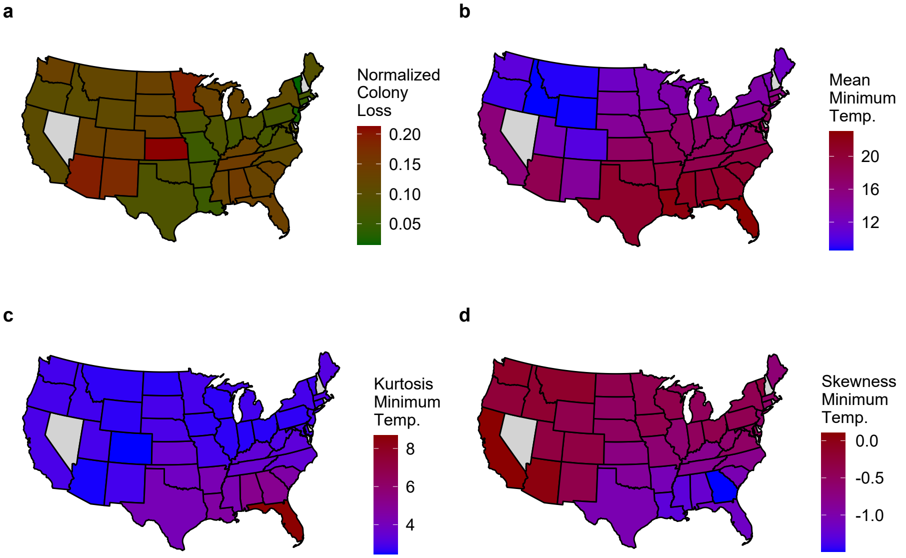

Fig. S13: Spatial representation of the median for third quarter data in the years 2015-2021. (a) Normalized colony loss. (b) Mean of minimum temperatures. (c) Kurtosis of minimum temperatures. (d) Skewness of minimum temperatures. The map has been generated by the authors in R 3.6.2 R Core Team (2021).

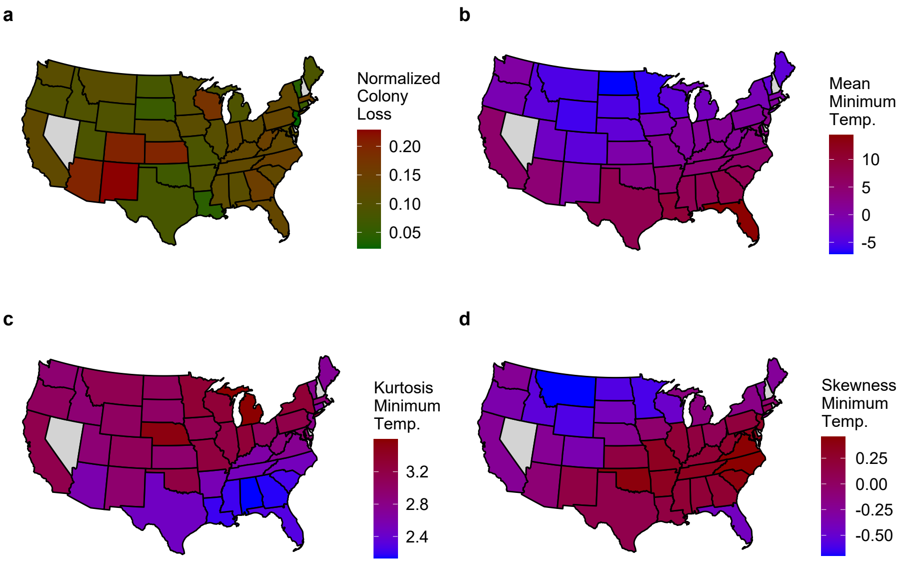

Fig. S14: Spatial representation of the median for fourth quarter data in the years 2015-2021. (a) Normalized colony loss. (b) Mean of minimum temperatures. (c) Kurtosis of minimum temperatures. (d) Skewness of minimum temperatures. The map has been generated by the authors in R 3.6.2 R Core Team (2021).

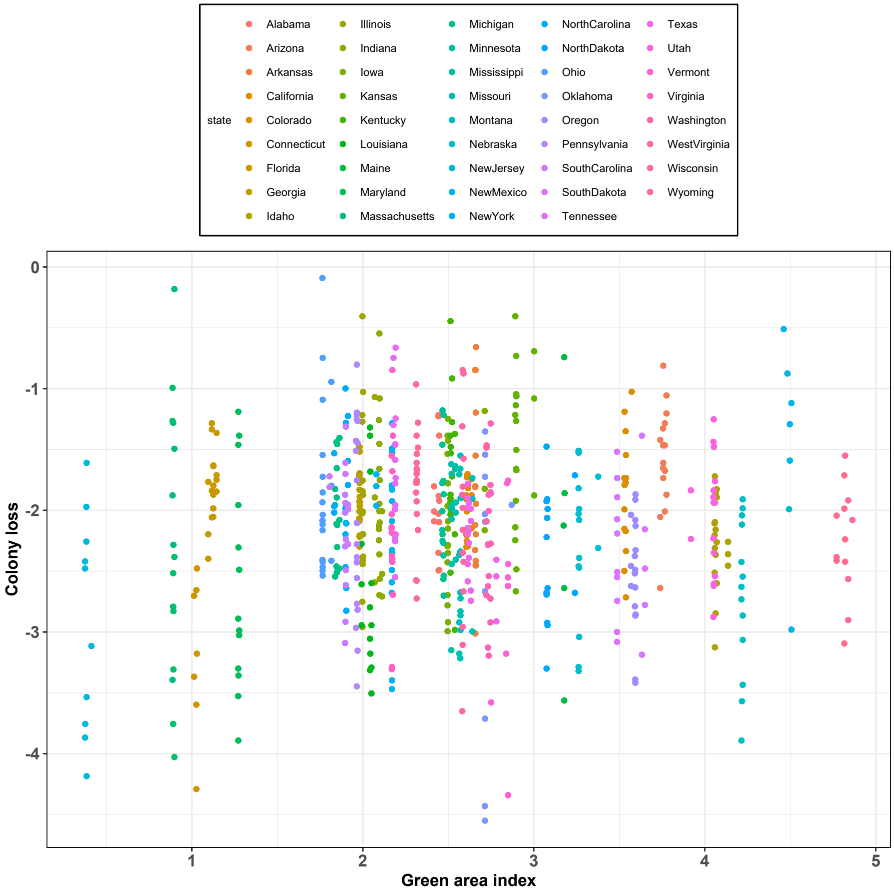

Fig. S15: Scatterplot of the response variable used in our linear modeling exercise against the transformed "green-area index" across each state in the United States for the years 2015-2019. Most states experience very little variability in terms of green areas across different years; hence, this feature is partially capturing state-level variability.

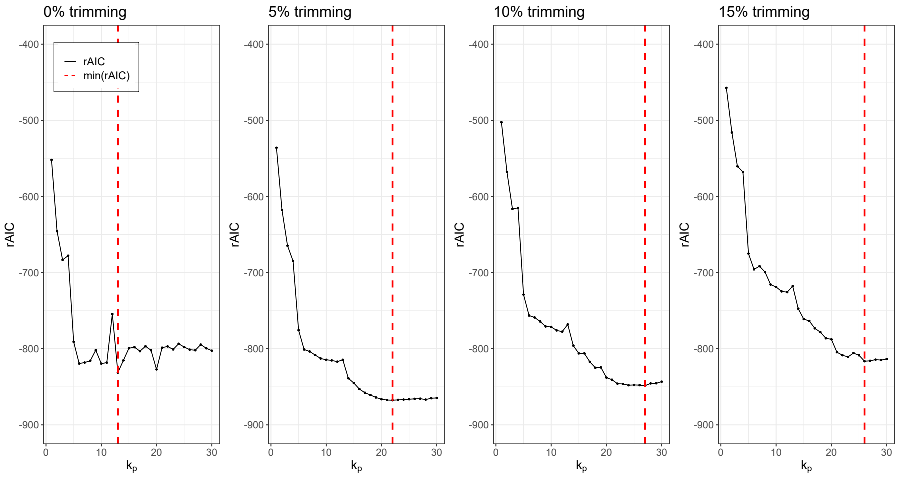

Fig. S16: Robust Akaike information criterion (rAIC; with minimum value indicated by a vertical dashed line) for our mixed-integer programming approach (Insolia et al., 2021) across sparsity levels and amounts of trimming. The sparsity level k_p increases from 1 to 30 with a step size of 1 (which is reported in the x-axis of each panel), and the trimming proportion k_n/n increases from 0% to 15% with a step size of 5% (from left to right). These results are based on data for the years 2015-2019.

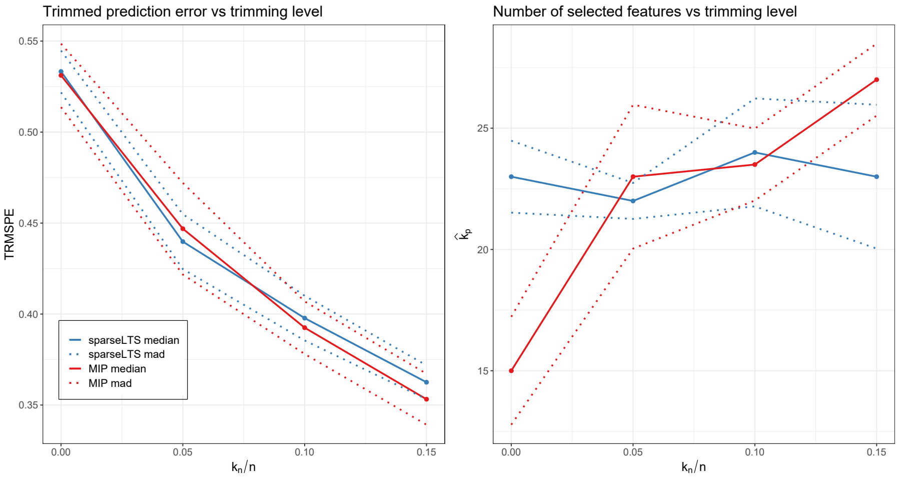

Fig. S17: Medians and medians ± median absolute deviations of the trimmed root mean squared prediction error (left panel) and number of selected features (right panel) for sparseLTS (Alfons et al., 2013) and our mixed-integer programming approach (Insolia et al., 2021). These are computed across trimming proportions k_n/n ranging from 0% to 15% (with a step size of 5%) and they are based on 8 independent training/testing splits containing 80% and 20% of the points, respectively. These results are based on data for the years 2015-2019.

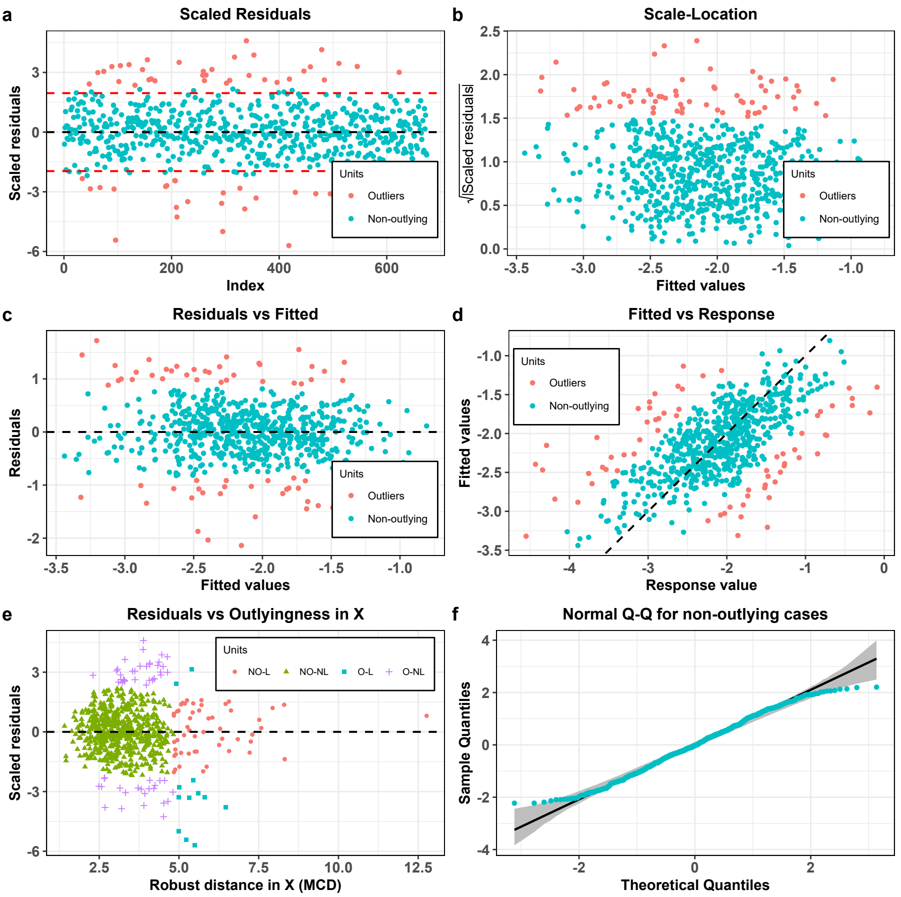

Fig. S18: Regression diagnostics for the model selected by our mixed-integer programming (MIP) approach (Insolia et al., 2021) with a 10% trimming. **(a)** Scaled residuals for outlying (red) and non-outlying (blue) cases. **(b)** Square root of absolute scaled residuals for outlying (red) and non-outlying (blue) cases. **(c)** Residuals for outlying (red) and non-outlying (blue) cases against fitted values (or predicted values for outlying cases). **(d)** Fitted values for non-outlying cases (blue) and predicted values for outlying cases (red) against the observed value of the response variable. **(e)** Scaled residuals against a robust measure of outlying-ness in the predictors' space. The latter is computed using the minimum covariance determinant estimator (Rousseeuw and Driessen, 1999) in the `rrcov` `R` package (Todorov and Filzmoser, 2009) where the trimming proportion is set to 10% as for MIP estimates and we used the 0.975 quantile of a chi-square distribution to flag leverage points (i.e., outliers in the predictors' space). Points are grouped as non-outlying cases and non-leverage points (NO-NL, green), outliers but non-leverage points (O-NL, purple), leverage points and non-outlying cases (NO-L, red), and outliers and leverage points (O-L, blue). **(f)** Normal QQ-plot for the scaled residuals of non-outlying cases. These results are based on data for the years 2015-2019.

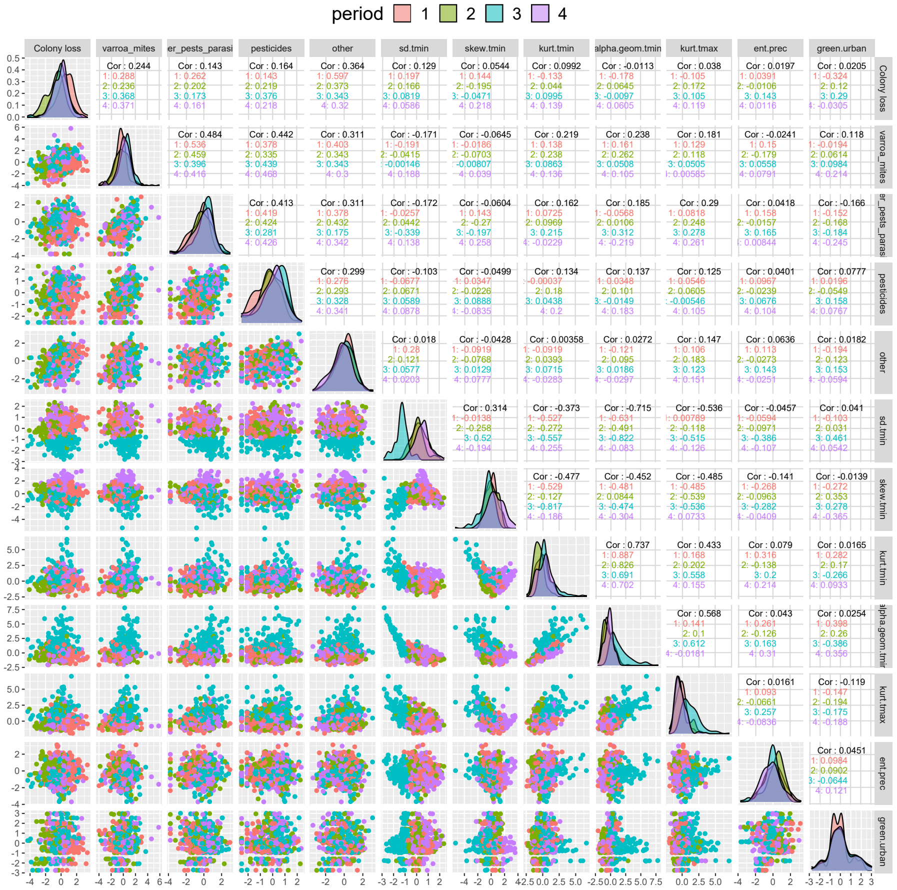

Fig. S19: Scatter matrix, marginal correlations and univariate densities across different quarters for the response variable and continuous predictors selected by our mixed-integer programming approach (Insolia et al., 2021) with a 10% trimming. These plots are based on data covering the years 2015-2019.

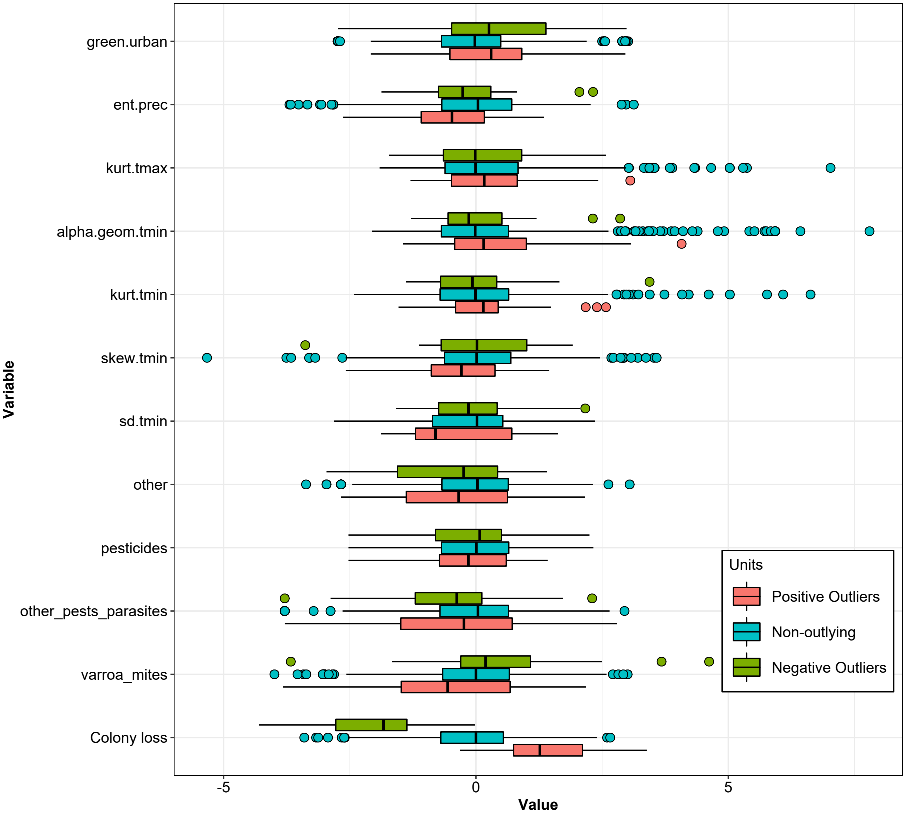

Fig. S20: Box plots for the response variable and continuous predictors selected by our mixed-integer programming approach (Insolia et al., 2021) with a 10% trimming, contrasting outlying and non-outlying case. Red, green and blue boxes represent points estimated as outliers with positive and negative residuals, and non-outlying cases, respectively. The values of each feature are scaled to have zero median and unit median absolute deviation. These results are based on data covering the years 2015-2019.

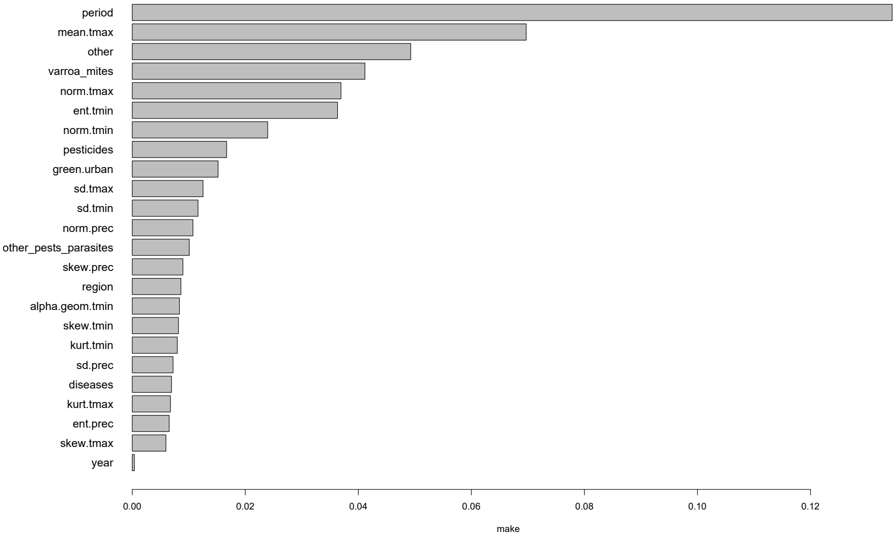

Fig. S21: Feature importance based on random forest (Breiman, 2001), which is computed through the `ranger` `R` package (Wright and Ziegler, 2017), for the same set of variables used in our linear modeling exercise. We compared an increasing number of trees (from 1000 to 5000 with a step size of 1000) and number of variables to possibly split at in each node (ranging from 5 to 15). Feature importance is based on a permutation approach (Wright et al., 2017). This result is based on data covering the years 2015-2019.

Table S1: Honey bee (*Apis mellifera*) colonies status and stressor data description. Adapted from the annual Honey Bee Colonies Loss Report released by the United States Department of Agriculture, National Agricultural Statistics Service (2022) – data downloaded from: https://usda.library.cornell.edu/concern/publications/rn301137d?locale=en. The data are collected by the National Agricultural Statistics Service through the Colony Loss Survey and recorded by states and quarters (January-March, April-June, July-September, October-December) for the years 2015-2021. Only operations with five or more total colonies are included in the survey, and beekeepers need to meet criteria on the definition of a farm (e.g., an agricultural product turnover higher than $1,000 per year). Data for Nevada, New Hampshire, Rhode Island and Delaware are not reported, as well as the second quarter of 2019. The questionnaire can be found at: https://www.nass.usda.gov/Publications/Methodology_and_Data_Quality/Honey_Bee_Colonies/index.php. A complete description of the methodology can be found in the Honey Bee Colonies Methodology and Quality Measures document, available at: https://www.nass.usda.gov/Publications/Methodology_and_Data_Quality/Honey_Bee_Colonies/10_2021/hbclqm21.pdf.

**Variable**	**Type**	**Description**
Maximum colonies	Count	Number of colonies on the first day of a quarter, plus all colonies that moved into that state during the quarter. Colonies that were added, lost, or renovated are not included, and colonies are counted in every state they were in during each quarter
Lost colonies	Count	Number of colonies no longer viable (e.g., completely failed colony, loss of most workers, and possibly the queen)
Percent lost	Percentage	Number of lost colonies divided by the maximum colonies
Added colonies	Count	Number of added colonies
Renovated colonies	Count	Number of surviving colonies that were requeened or received new honey bees through nuc or package
Percent renovated	Percentage	Number of renovated colonies divided by the maximum colonies
Varroa destructor	Percentage	Number of colonies affected by *Varroa destructor* divided by the maximum colonies
Other pests and parasites	Percentage	Number of colonies affected by tracheal mites, nosema, hive beetle, wax moths, etc. divided by the maximum colonies
Diseases	Percentage	Number of colonies affected by certain diseases divided by the maximum colonies. Diseases include American and European foulbrood, chalkbrood, stonebrood, paralysis (acute and chronic), kashmir, deformed wing, sacbrood, IAPV, Lake Sinai II, etc.
Pesticides	Percentage	Number of colonies affected by pesticides divided by the maximum colonies
Other	Percentage	Number of colonies affected by other events divided by the maximum colonies. These events include weather, starvation, insufficient forage, queen failure, hive damage/destroyed, etc.
Unknown	Percentage	Number of colonies affected by unknown causes divided by the maximum colonies

Table S2: Weather indexes description. The weather indexes that we built are based on Parameter-elevation Regressions on Independent Slopes Model (PRISM) data (PRISM Climate Group, 2022) – raw data downloaded from https://prism.oregonstate.edu/. The PRISM 4-kilometer-squared gridded daily temperature and precipitation data were used to generate weather-related variables covering 2015-2021 and the whole United States (by states and quarters). For each combination of day and element of the grid, the maximum and minimum temperature were extracted, as well as total precipitation (given by the combination of rain and melted snow). See the *Data processing* Section of the main text for further details on the computation of these indexes.

**Variable**	**Description**
Min./Max. temp. mean	Mean minimum and mean maximum temperature
Min./Max. temp. std. dev.	Standard deviation of the minimum and maximum temperature
Min./Max. temp. norm	L_2-norm for minimum and maximum temperature
Min./Max. temp. entropy	Entropy for minimum and maximum temperature
Min./Max. temp. skewness	Skewness of the minimum and maximum temperature
Min./Max. temp. kurtosis	Kurtosis of the minimum and maximum temperature
Min./Max. temp. alpha index	Tail exponent α of the minimum and maximum temperature
Precipitation mean	Mean precipitation
Precipitation std. dev.	Standard deviation of precipitation
Precipitation norm	L_2-norm of precipitation
Precipitation entropy	Entropy of precipitation
Precipitation skewness	Skewness of precipitation
Precipitation kurtosis	Kurtosis of precipitation
Precipitation alpha index	Tail exponent α of precipitation

Table S3: Land use data description. Adapted from the Cropland Data Layer (CDL) which is provided by the National Agricultural Statistics Service of the United States Department of Agriculture (Boryan et al., 2011) – data downloaded from https://www.nass.usda.gov/Research_and_Science/Cropland/sarsfaqs2.php. CDL data are collected annually and cover the whole United States at the resolution of a 30-meter-squared grid. They were used to generate annual land-use data for the years 2015-2021 across states of the United States. The original land-use categories were grouped in 6 major classes: "developed", "forest", "pasture", "rangeland", "crop", and "water". Following the approach in Naug (2009), the "water" class was excluded from our analysis.

**Variables**	**Group**
Developed	Developed, Developed/High Intensity, Developed/Low Intensity, Developed/Medium Intensity, Developed/Open Space
Forest	Deciduous Forest, Evergreen Forest, Mixed Forest, Forest
Pasture	Grassland/Pasture, Pasture/Grass
Rangeland	Nonag/Undefined, Shrubland, Sod/Grass Seed, Herbs
Crop	Alfalfa, Almonds, Apples, Apricots, Aquaculture, Asparagus, Barley, Barren, Blueberries, Broccoli, Buckwheat, Cabbage, Camelina, Caneberries, Canola, Cantaloupes, Carrots, Cauliflower, Celery, Cherries, Chick Peas, Christmas Trees, Citrus, Clover/Wildflowers, Corn, Cotton, Cranberries, Cucumbers, Dbl Crop Barley/Corn, Dbl Crop Barley/Sorghum, Dbl Crop Barley/Soybeans, Dbl Crop Corn/Soybeans, Dbl Crop Durum Wht/Sorghum, Dbl Crop Lettuce/Barley, Dbl Crop Lettuce/Cantaloupe, Dbl Crop Lettuce/Cotton, Dbl Crop Lettuce/Durum Wht, Dbl Crop Oats/Corn, Dbl Crop Soybeans/Cotton, Dbl Crop Soybeans/Oats, Dbl Crop WinWht/Corn, Dbl Crop WinWht/Cotton, Dbl Crop WinWht/Sorghum, Dbl Crop WinWht/Soybeans, Dry Beans, Durum Wheat, Eggplants, Fallow/Idle Cropland, Flaxseed, Garlic, Gourds, Grapes, Greens, Honeydew Melons, Hops, Lentils, Lettuce, Millet, Mint, Misc Vegs & Fruits, Mustard, Nectarines, Oats,Olives, Onions, Oranges, Other Crops, Other Hay/Non Alfalfa, Other Small Grains, Other Tree Crops, Peaches, Peanuts, Pears, Peas, Pecans, Peppers, Pistachios, Plums, Pomegranates, Pop or Orn Corn, Potatoes, Prunes, Pumpkins, Radishes, Rape Seed, Rice, Rye, Safflower, Sorghum, Soybeans, Speltz, Spring Wheat, Squash, Strawberries, Sugarbeets, Sugarcane, Sunflower, Sweet Corn, Sweet Potatoes, Switchgrass, Tobacco, Tomatoes, Triticale, Turnips, Vetch, Walnuts, Watermelons, Winter Wheat
Water	Herbaceous Wetlands, Open Water, Perennial Ice/Snow, Water, Wetlands, Woody Wetlands

Table S4: Explanatory variables included in our linear modeling exercise (excluding the intercept term) for the years 2015-2019. For each variable we provide information regarding its nature before any possible transformation (e.g., percentage, count, continuous or binary), as well a reference to the dataset it refers to (i.e., a table or figure where it is discussed in more detail).

#	Variable	Type	Reference
1	Year 2015	binary	Tables S1, S2, S3
2	Year 2016	binary	Tables S1, S2, S3
3	Year 2017	binary	Tables S1, S2, S3
4	Year 2018	binary	Tables S1, S2, S3
5	Year 2019	binary (reference category)	Tables S1, S2, S3
6	Region Central	binary (reference category)	Figure S1
7	Region East North Central	binary	Figure S1
8	Region Northeast	binary	Figure S1
9	Region Northwest	binary	Figure S1
10	Region South	binary	Figure S1
11	Region Southeast	binary	Figure S1
12	Region Southwest	binary	Figure S1
13	Region West	binary	Figure S1
14	Region West North Central	binary	Figure S1
15	Quarter 1	binary	Tables S1, S2
16	Quarter 2	binary	Tables S1, S2
17	Quarter 3	binary	Tables S1, S2
18	Quarter 4	binary (reference category)	Tables S1, S2
19	*Varroa destructor*	percentage	Table S1
20	Other pests and parasites	percentage	Table S1
21	Diseases	percentage	Table S1
22	Pesticides	percentage	Table S1
23	Other	percentage	Table S1
24	Min. temp. std. dev.	continuous	Table S2
25	Min. temp. norm	continuous	Table S2
26	Min. temp. entropy	continuous	Table S2
27	Min. temp. skewness	continuous	Table S2
28	Min. temp. kurtosis	continuous	Table S2
29	Min. temp. alpha index	continuous	Table S2
30	Max. temp. mean	continuous	Table S2
31	Max. temp. std. dev.	continuous	Table S2
32	Max. temp. norm	continuous	Table S2
33	Max. temp. skewness	continuous	Table S2
34	Max. temp. kurtosis	continuous	Table S2
35	Precipitation std. dev.	continuous	Table S2
36	Precipitation norm	continuous	Table S2
37	Precipitation entropy	continuous	Table S2
38	Precipitation skewness	continuous	Table S2
39	Green-area index	continuous	Table S3

Table S5: F-tests (Hastie and Pregibon, 1992) on the contribution of weather indexes for the years 2015-2019. Comparisons across nested fits for the Gaussian – both with and without the 10% outliers detected by our mixed-integer programming approach (Insolia et al., 2021) – and quasi-Poisson models: sample size (n), number of features (p), residual degree of freedom and residual deviance (residual sum of squares for the Gaussian models), difference in the degree of freedom between nested models, deviance (sum of squares for the Gaussian case), F-statistic, and p-value. For minimum and maximum temperatures, as well as precipitation, the associated reduced models exclude the following weather indexes that we built: "norm", "entropy", "skewness", "kurtosis", and "alpha index".

**Model**	n	p	**Resid. Df**	**Resid. Dev**	**Df**	**Dev**	F	**Pr(> F)**
Gaussian	674	37	637	168.78				
(with MIP outliers)	674	26	648	174.20	-11	-5.42	1.86	0.0417
Gaussian	607	37	570	80.37				
(without MIP outliers)	607	26	581	86.79	-11	-6.42	4.14	$< 10^{-4}$
Quasi-Poisson	674	37	637	618935.58				
(with MIP outliers)	674	26	648	643098.12	-11	-24162.54	2.17	0.0144
Quasi-Poisson	674	37	570	348561.16				
(without MIP outliers)	674	26	581	371029.26	-11	-22468.10	3.30	0.0002

Table S6: Spatio-temporal information for outlying cases with unexpectedly higher or lower losses (i.e., large positive or negative residuals, respectively) detected by our mixed-integer programming approach (Insolia et al., 2021) with a 10% trimming for the years 2015-2019. We consider scaled residuals exceeding a given quantile of the standard normal distribution (first column), and for these points we report the number of observations belonging to each climatic region, state, year and quarter.

Quantile	Region	# Obs.	State	# Obs.	Year	# Obs.	Quarter	# Obs.
$> \mathbf{\Phi}^{-1}(0.975)$	South	11	Kansas	5	2015	9	1	5
	West North Central	9	Nebraska	4	2016	7	2	6
	Central	5	Arkansas	3	2017	7	3	16
	Northeast	5	Massachusetts	3	2018	8	4	8
	Northwest	3	South Dakota	3	2019	4		
	Southeast	1	Washington	3				
	(Other)	1	(Other)	14				
$> \mathbf{\Phi}^{-1}(0.999)$ X	South	4	Kansas	2	2015	4	1	3
	Central	3	South Dakota	2	2016	2	2	2
	Northeast	3	Arkansas	1	2017	4	3	9
	West North Central	3	Illinois	1	2018	1	4	0
	Northwest	1	Louisiana	1	2019	3		
	East North Central	0	Maine	1				
	(Other)	0	(Other)	6				
$< \mathbf{\Phi}^{-1}(0.025)$	Northeast	9	Vermont	4	2015	9	1	6
	South	7	Oklahoma	3	2016	9	2	7
	Southwest	4	Louisiana	2	2017	5	3	9
	Central	3	New Jersey	2	2018	3	4	10
	Northwest	3	Oregon	2	2019	6		
	East North Central	2	Utah	2				
	(Other)	4	(Other)	17				
$< \mathbf{\Phi}^{-1}(0.001)$	Northeast	6	Oklahoma	2	2015	6	1	2
	South	4	Vermont	2	2016	3	2	3
	Central	2	Arkansas	1	2017	3	3	6
	Southwest	2	Connecticut	1	2018	1	4	4
	Northwest	1	Idaho	1	2019	2		
	East North Central	0	Kentucky	1				
	(Other)	0	(Other)	7				

Table S7: Regression results for the colony loss response against the sole predictor "other pests and parasites" (Model 1), against the sole predictor *V. destructor* (Model 2), against both "other pests and parasites" and *V. destructor* (Model 3), and finally against these two predictors and "other" (Model 4) for the years 2015-2019. For each predictor in each fit, we report the corresponding coefficient estimate, standard error, t-statistic and p-value. Outlying cases detected by our mixed-integer programming approach (Insolia et al., 2021) based on a 10% trimming were excluded from the analysis. While the marginal regressions in Models 1-2 have positive coefficients, the coefficient for "other pests and parasites" changes sign in Model 3, and becomes negative and significant after the inclusion of the variable "other" in Model 4.

**Model**	**Coefficient**	**Estimate**	**Std. Error**	t **value**	**Pr(> |t|)**
1	(Intercept)	-1.9608	0.0498	-39.34	$< 2 \times 10^{-16}$
	Other pests and parasites	0.0564	0.0180	3.14	0.0018
2	(Intercept)	-1.9348	0.0294	-65.77	$< 2 \times 10^{-16}$
	Varroa destructor	0.1957	0.0229	8.54	$< 2 \times 10^{-16}$
3	(Intercept)	-1.9672	0.0475	-41.43	$< 2 \times 10^{-16}$
	Other pests and parasites	-0.0169	0.0195	-0.87	0.3853
	Varroa destructor	0.2064	0.0260	7.92	1.11×10^{-14}
4	(Intercept)	-1.5086	0.0686	-22.00	$< 2 \times 10^{-16}$
	Other pests and parasites	-0.0450	0.0186	-2.42	0.0158
	Varroa destructor	0.1555	0.0252	6.17	1.25×10^{-9}
	Other	0.1976	0.0224	8.82	$< 2 \times 10^{-16}$

Table S8: Comparison of estimated regression coefficients' signs across different estimation methods and models (negative/positive signs are reported as red/green cells) for the years 2015-2019. For our linear modeling exercise, as the one used by our mixed-integer programming (MIP) approach (Insolia et al., 2021) in the main text, we consider: *OLS*: ordinary least squares; *glmnet*: elastic-net penalty with mixing parameter $\alpha = 0.8$ (Simon et al., 2011); *SCAD*: smoothly-clipped absolute deviations penalty (Breheny and Huang, 2011); *sparseLTS*: lasso penalty with least trimmed of squares loss based on a 10% trimming (Alfons, 2021). For the same model using count data as a response variable (i.e., the number of lost colonies per state and quarter) and an additional offset to capture different scales (i.e., the logarithm of maximum number of colonies per state and quarter), we compare: *glmnet-Poisson*: elastic-net penalty for Poisson models with mixing parameter $\alpha = 0.8$ (Simon et al., 2011); *snet-NB*: SCAD penalty with a ridge-like parameter (also here we set $\alpha = 0.8$) for negative binomial models (Breheny and Huang, 2011). Overall, these results are fairly consistent across different methods, and with the ones discussed in the main text for MIP. However, MIP provides a sparser and more interpretable solution.

Coefficient	OLS	glmnet	SCAD	sparseLTS	glmnet-Poisson	snet-NB
(Intercept)	−	−	−	−	−	−
Year 2015	+	+		+	+	+
Year 2016	+	−				−
Year 2017	+				−	
Year 2018	+				+	
East North Central	−	−		−	+	−
Northeast	−	−	−	−		−
Northwest	−	−	−	−	−	−
South	−				−	
Southeast	+			+		
Southwest	+	+	+	+	+	+
West	−	−		−		−
West North Central	−	−	−	−	−	−
Quarter 1	+	+	+	+	+	+
Quarter 2	−	−	−	−	−	−
Quarter 3	+					
Varroa destructor	+	+	+	+	+	+
Other pests and parasites	−			−		−
Diseases	+					
Pesticides	+	+	+	+	+	+
Other	+	+	+	+	+	+
Min. temp. std. dev.	+	+	+	+		+
Min. temp. norm	−					
Min. temp. entropy	+				−	
Min. temp. skewness	−	+		+	−	
Min. temp. kurtosis	+	+		+		+
Min. temp. alpha index	−			−	−	
Max. temp. mean	−	−		−		
Max. temp. std. dev.	−				−	
Max. temp. norm	+					
Max. temp. skewness	+	+				+
Max. temp. kurtosis	+			+	+	
Precipitation std. dev.	+				+	
Precipitation norm	−					
Precipitation entropy	+	+		+	−	+
Precipitation skewness	−				−	
Green-area index	−			+	−	+

Table S9: Comparison of regression coefficients' signs (negative/positive signs are reported as red/green cells) across different estimation methods and models as in Table S8 for the years 2015-2019. Here each fit is computed on the set of non-outlying cases detected by our mixed-integer programming (MIP) approach (Insolia et al., 2021) with a 10% trimming. In this setting, results are more consistent across methods and models, and they resemble more closely the ones discusses in the main text based on MIP.

Coefficient	OLS	glmnet	SCAD	sparseLTS	glmnet-Poisson	snet-NB
(Intercept)	red	red	red	red	red	red
Year 2015	green	green	green	green	green	green
Year 2016	green	green			green	
Year 2017	green			green	red	
Year 2018	red	red		red	green	red
East North Central	red	red	red	red	green	red
Northeast	red	red	red	red		red
Northwest	red	red	red	red	red	red
South	red	red	red		red	red
Southeast	green	green	green	green		green
Southwest	red	green	green	green	green	
West	red	red	red	red	red	red
West North Central	red	red	red	red	red	red
Quarter 1	green	green	green	green	green	green
Quarter 2	red	red	red	red	red	red
Quarter 3	green				red	
Varroa destructor	green	green	green	green	green	green
Other pests and parasites	red	red	red	red	red	red
Diseases	red				green	
Pesticides	green	green	green	green		green
Other	green	green	green	green	green	green
Min. temp. std. dev.	green	green	green	green		green
Min. temp. norm	red					
Min. temp. entropy	red					
Min. temp. skewness	green	green	green	green		green
Min. temp. kurtosis	green	green	green	green	green	green
Min. temp. alpha index	red	red	red	red	red	red
Max. temp. mean	red	red	red	red	red	
Max. temp. std. dev.	red					
Max. temp. norm	green					
Max. temp. skewness	red					
Max. temp. kurtosis	green	green	green	green	green	green
Precipitation std. dev.	green				green	
Precipitation norm	red					
Precipitation entropy	green	green	green	green		green
Precipitation skewness	green				red	
Green-area index	green	green	green	green		green

Table S10: Features selected by our mixed-integer programming approach (Insolia et al., 2021) with a 10% trimming, for the years 2015-2019, using state-level controls as opposed to climatic regions. The results are consistent with the ones discussed in the main text, with the difference that the "green-area index" switches sign and results as non-significant, as it partially accounts for state-level variability (see Figure S15). Model with $R^2 = 0.65$.

Coefficient	Estimate	Std. Error	t value	Pr($>|t|$)
(Intercept)	-1.7738	1.2278	-1.44	0.1491
Arizona	0.1094	0.6708	0.16	0.8705
Arkansas	-0.1449	0.1861	-0.78	0.4366
California	-0.4107	0.1659	-2.48	0.0136
Colorado	-0.1148	0.5634	-0.20	0.8387
Connecticut	-0.8277	0.7260	-1.14	0.2548
Florida	-0.1767	0.6687	-0.26	0.7916
Georgia	-0.2331	0.2654	-0.88	0.3801
Idaho	-0.4450	0.8212	-0.54	0.5881
Illinois	-0.1902	0.2538	-0.75	0.4541
Indiana	-0.2053	0.2186	-0.94	0.3480
Iowa	-0.4332	0.1438	-3.01	0.0027
Kansas	0.0145	0.2774	0.05	0.9583
Kentucky	-0.0120	0.1416	-0.08	0.9327
Louisiana	-0.7142	0.2495	-2.86	0.0044
Maine	-0.1318	0.4264	-0.31	0.7573
Maryland	-0.5815	0.5958	-0.98	0.3295
Massachusetts	-0.6169	0.7822	-0.79	0.4307
Michigan	-0.3856	0.3301	-1.17	0.2432
Minnesota	-0.1950	0.1465	-1.33	0.1836
Mississippi	-0.2392	0.1645	-1.45	0.1463
Missouri	-0.4262	0.1529	-2.79	0.0055
Montana	-0.6731	0.9057	-0.74	0.4577
Nebraska	-0.5472	0.4478	-1.22	0.2223
New Jersey	-0.7514	1.0338	-0.73	0.4677
New Mexico	0.5507	1.0350	0.53	0.5949
New York	-0.4051	0.2001	-2.02	0.0434
North Carolina	-0.2622	0.3058	-0.86	0.3916
North Dakota	-0.4851	0.3613	-1.34	0.1800
Ohio	-0.2840	0.3602	-0.79	0.4308
Oklahoma	0.0389	0.2360	0.16	0.8693
Oregon	-0.6303	0.5853	-1.08	0.2820
Pennsylvania	-0.2796	0.2742	-1.02	0.3082
South Carolina	-0.2622	0.3031	-0.86	0.3875
South Dakota	-0.6196	0.5678	-1.09	0.2757
Tennessee	0.0044	0.1884	0.02	0.9814
Texas	-0.3294	0.1684	-1.96	0.0509
Utah	0.0866	0.8043	0.11	0.9143
Vermont	-0.1514	0.2965	-0.51	0.6097
Virginia	-0.2159	0.1976	-1.09	0.2751
Washington	-0.5987	0.2100	-2.85	0.0045
West Virginia	-0.1110	0.1650	-0.67	0.5012
Wisconsin	-0.2413	0.1517	-1.59	0.1122
Wyoming	-0.1348	1.1873	-0.11	0.9096
Year 2016	-0.1228	0.0485	-2.53	0.0116
Year 2017	-0.1347	0.0477	-2.82	0.0049
Year 2018	-0.1526	0.0503	-3.03	0.0025
Year 2019	-0.1352	0.0568	-2.38	0.0178
Quarter 2	-0.7523	0.0489	-15.39	$< 10^{-4}$
Quarter 3	-0.3639	0.0756	-4.82	$< 10^{-4}$
Quarter 4	-0.4088	0.0476	-8.58	$< 10^{-4}$
Varroa destructor	0.1743	0.0221	7.90	$< 10^{-4}$
Other pests and parasites	-0.0711	0.0169	-4.20	$< 10^{-4}$
Pesticides	0.0223	0.0127	1.75	0.0802
Other	0.1578	0.0181	8.71	$< 10^{-4}$
Min. temp. std. dev.	0.0557	0.0208	2.68	0.0076
Min. temp. skewness	0.1861	0.0497	3.74	0.0002
Min. temp. kurtosis	0.5509	0.1196	4.60	$< 10^{-4}$
Min. temp. alpha index	-0.2016	0.0735	-2.74	0.0063
Max. temp. kurtosis	0.2344	0.1097	2.14	0.0331
Precipitation entropy	0.0710	0.0351	2.02	0.0435
Green-area index	-0.0645	0.4965	-0.13	0.8967

Table S11: Ordinary least squares fit computed on the sets of non-outlying cases and selected features by our mixed-integer programming (MIP) approach (Insolia et al., 2021) based on a 10% trimming, for the years 2015-2019, with additional lagged variables for stressors and weather indexes. Lags are computed according to data for the previous quarter (if available). The model contains $p = 25$ predictors (including the intercept term), and after the removal of outliers detected by MIP and missing data the sample size reduces to $n = 437$. For each predictor, we report the corresponding coefficient estimate, standard error, t-statistic and p-value. The model has an $R^2 = 0.58$. The results are consistent with the ones discussed in the main text for MIP, and only a few lagged terms report a significant coefficient; namely: "lagged *V. destructor*", "lagged skewness of minimum temperatures", and "lagged kurtosis of minimum temperatures".

**Coefficient**	**Estimate**	**Std. Error**	t **value**	**Pr(>** $\|t\|$**)**
(Intercept)	-3.4644	0.4066	-8.52	$< 10^{-4}$
Region East North Central	-0.1574	0.0720	-2.19	0.0292
Region Northeast	-0.2360	0.0702	-3.36	0.0009
Region Northwest	-0.6493	0.1083	-5.99	$< 10^{-4}$
Region South	-0.2815	0.0828	-3.40	0.0007
Region Southeast	0.0142	0.0655	0.22	0.8290
Region Southwest	-0.1416	0.1246	-1.14	0.2565
Region West	-0.4405	0.1270	-3.47	0.0006
Region West North Central	-0.5165	0.1130	-4.57	$< 10^{-4}$
Year 2015	0.1750	0.0830	2.11	0.0356
Year 2016	0.0618	0.0763	0.81	0.4183
Year 2017	0.0472	0.0743	0.64	0.5255
Year 2018	0.0141	0.0755	0.19	0.8521
Quarter 1	0.2986	0.0903	3.31	0.0010
Quarter 2	-0.3394	0.0889	-3.82	0.0002
Quarter 3	-0.0040	0.1030	-0.04	0.9689
Varroa destructor	0.1826	0.0257	7.11	$< 10^{-4}$
Other pests and parasites	-0.0664	0.0195	-3.40	0.0007
Pesticides	0.0400	0.0150	2.67	0.0079
Other	0.1278	0.0216	5.93	$< 10^{-4}$
Min. temp. std. dev.	0.0324	0.0231	1.40	0.1622
Min. temp. skewness	0.1887	0.0606	3.11	0.0020
Min. temp. kurtosis	0.6139	0.1455	4.22	$< 10^{-4}$
Min. temp. alpha index	-0.2574	0.0989	-2.60	0.0096
Max. temp. kurtosis	0.2826	0.1245	2.27	0.0238
Precipitation entropy	0.0899	0.0365	2.46	0.0143
Green-area index	0.1056	0.0463	2.28	0.0231
Lagged *Varroa destructor*	0.0472	0.0261	1.81	0.0718
Lagged Other pests and parasites	-0.0071	0.0196	-0.36	0.7187
Lagged Pesticides	0.0001	0.0150	0.01	0.9956
Lagged Other	-0.0270	0.0219	-1.23	0.2191
Lagged Min. temp. std. dev.	0.0349	0.0257	1.36	0.1744
Lagged Min. temp. skewness	0.1447	0.0624	2.32	0.0209
Lagged Min. temp. kurtosis	0.3927	0.1404	2.80	0.0054
Lagged Min. temp. alpha index	-0.0527	0.0861	-0.61	0.5404
Lagged Max. temp. kurtosis	0.1133	0.1224	0.93	0.3550
Lagged Precipitation entropy	-0.0349	0.0356	-0.98	0.3273

Table S12: Ordinary least squares fit for the features selected by glmnet (Simon et al., 2011) – with mixing parameter $\alpha = 0.8$ and enforcing the inclusion of features selected by our mixed-integer programming (MIP) approach (Insolia et al., 2021) – for a model as in Table 1 of the main text, covering the years 2015-2019, with 55 additional pairwise interaction terms computed among continuous features only ($p = 82$). Interaction terms with a marginal correlation larger than 0.7 were removed at the outset (as described in the *Data treatment* Section of the Supplementary Information), reducing the number of predictors to $p = 48$. Outlying cases detected by our MIP based on a 10% trimming were excluded from the analysis ($n = 607$). The model has an $R^2 = 0.62$. Most predictors selected by MIP remain significant, such as "*V. destructor*", "pesticides", "kurtosis of maximum temperatures", "entropy of precipitations", "green-area index", etc. The interaction terms with a significant coefficient include the interaction of the "green-area index" with "other", "standard deviation of minimum temperatures", "entropy of precipitations", and "skewness of minimum temperature", as well as the interaction of the "alpha index of minimum temperatures" with "*V. destructor*" and "standard deviation of minimum temperatures". Overall, these results are consistent with the ones based on MIP which are discussed in the main text.

**Coefficient**	**Estimate**	**Std. Error**	***t* value**	**Pr(> |*t*|)**
(Intercept)	-4.4525	0.4915	-9.06	$< 10^{-4}$
Year 2015	0.1205	0.0563	2.14	0.0329
Year 2016	0.0199	0.0550	0.36	0.7177
Year 2017	0.0161	0.0540	0.30	0.7658
Year 2018	-0.0295	0.0538	-0.55	0.5837
Region East North Central	-0.1733	0.0609	-2.85	0.0046
Region Northeast	-0.1210	0.0611	-1.98	0.0480
Region Northwest	-0.5910	0.0859	-6.88	$< 10^{-4}$
Region South	-0.1448	0.0641	-2.26	0.0242
Region Southeast	0.0197	0.0569	0.35	0.7290
Region Southwest	-0.0464	0.0943	-0.49	0.6225
Region West	-0.2023	0.1088	-1.86	0.0634
Region West North Central	-0.5694	0.0856	-6.65	$< 10^{-4}$
Quarter 1	0.3942	0.0480	8.22	$< 10^{-4}$
Quarter 2	-0.3772	0.0532	-7.10	$< 10^{-4}$
Quarter 3	0.0023	0.0750	0.03	0.9758
Varroa destructor	0.2510	0.0372	6.75	$< 10^{-4}$
Other pests and parasites	-0.1911	0.0735	-2.60	0.0096
Pesticides	0.0303	0.0122	2.49	0.0130
Other	0.0860	0.1003	0.86	0.3915
Min. temp. std. dev.	0.1822	0.0456	4.00	0.0001
Min. temp. skewness	-0.1940	0.1506	-1.29	0.1982
Min. temp. kurtosis	0.5573	0.1240	4.49	$< 10^{-4}$
Min. temp. alpha index	-0.1040	0.1081	-0.96	0.3365
Max. temp. kurtosis	0.8358	0.2693	3.10	0.0020
Precipitation entropy	0.2311	0.0838	2.76	0.0060
Green-area index	0.4456	0.1263	3.53	0.0005
Varroa destructor:Min. temp. skewness	-0.0392	0.0502	-0.78	0.4347
Varroa destructor:Min. temp. alpha index	-0.1002	0.0571	-1.75	0.0799
Other pests and parasites:Min. temp. alpha index	0.0346	0.0446	0.78	0.4381
Other pests and parasites:Max. temp. kurtosis	0.0869	0.0773	1.12	0.2616
other:Max. temp. kurtosis	0.1298	0.0836	1.55	0.1211
Other:Green-area index	-0.0285	0.0171	-1.67	0.0958
Min. temp. std. dev.:Min. temp. alpha index	-0.0411	0.0248	-1.65	0.0988
Min. temp. std. dev.:Green-area index	-0.0433	0.0143	-3.02	0.0026
Min. temp. skewness:Green-area index	0.1303	0.0546	2.39	0.0174
Precipitation entropy:Green-area index	-0.0530	0.0285	-1.86	0.0638

Table S13: Features selected using the mixed-integer programming procedure described in Insolia et al. (2021) for the years 2015-2021, with corresponding coefficient estimates, standard errors, t-statistics and p-values computed on a subset encompassing 90% of the observations (745 out of 828 are selected as "non-outlying", concurrently with the feature selection). Group-constraints are used to ensure that categorical controls for quarter and climatic region, e.g., the three terms representing quarters, are either all selected or all excluded. Moreover, we imposed the control for years to be retained in the model – as this would at least partially mitigate the effects of any anomalies in the last two years on the overall fit (here the reference category is the year 2021). Overall, this extended analysis confirms the main findings from Table 1 of the main text. The model has an $R^2 = 0.56$.

**Coefficient**	**Estimate**	**Std. Error**	***t* value**	**Pr(> |*t*|)**
(Intercept)	-2.2583	0.2173	-10.39	$< 10^{-4}$
Year 2015	0.1306	0.0596	2.19	0.0288
Year 2016	0.0234	0.0596	0.39	0.6952
Year 2017	0.0404	0.0592	0.68	0.4949
Year 2018	0.0382	0.0589	0.65	0.5167
Year 2019	0.0022	0.0647	0.03	0.9726
Year 2020	-0.0728	0.0679	-1.07	0.2841
Region East North Central	-0.2090	0.0630	-3.32	0.0010
Region Northeast	-0.2157	0.0585	-3.69	0.0002
Region Northwest	-0.7430	0.0781	-9.51	$< 10^{-4}$
Region South	-0.0870	0.0647	-1.34	0.1795
Region Southeast	0.1219	0.0589	2.07	0.0391
Region Southwest	-0.0616	0.0918	-0.67	0.5027
Region West	-0.2881	0.1026	-2.81	0.0051
Region West North Central	-0.5780	0.0799	-7.23	$< 10^{-4}$
Quarter 1	0.2691	0.0505	5.33	$< 10^{-4}$
Quarter 2	-0.2607	0.0704	-3.71	0.0002
Quarter 3	0.0377	0.1031	0.37	0.7147
Varroa destructor	0.1711	0.0196	8.74	$< 10^{-4}$
Other pests and parasites	-0.0682	0.0157	-4.33	$< 10^{-4}$
Pesticides	0.0258	0.0116	2.23	0.0260
Other	0.1858	0.0163	11.37	$< 10^{-4}$
Min. temp. skewness	0.1053	0.0461	2.28	0.0227
Min. temp. kurtosis	0.4844	0.1083	4.47	$< 10^{-4}$
Min. temp. alpha index	-0.2571	0.0620	-4.15	$< 10^{-4}$
Max. temp. mean	-0.0129	0.0056	-2.31	0.0212
Max. temp. kurtosis	0.1535	0.0939	1.64	0.1024
Precipitation entropy	0.0764	0.0332	2.30	0.0215
Precipitation skewness	0.0157	0.0079	1.98	0.0477
Green-area index	0.1527	0.0333	4.59	$< 10^{-4}$

Data

The Supplementary Information includes the dataset that we built covering the years 2015-2021 in the `bee_data.csv` file.

Source code

The Supplementary Information includes the source code to reproduce our analyses in the `code.zip` file.

References

Alfons, A. 2021. robustHD: An R package for robust regression with high-dimensional data. *Journal of Open Source Software 6*(67): 3786. https://doi.org/10.21105/joss.03786 .

Alfons, A., C. Croux, and S. Gelper. 2013. Sparse least trimmed squares regression for analyzing high-dimensional large data sets. *The Annals of Applied Statistics 7*(1): 226–248. https://doi.org/10.1214/12-AOAS575 .

Boryan, C., Z. Yang, R. Mueller, and M. Craig. 2011. Monitoring US agriculture: The US Department of Agriculture, National Agricultural Statistics Service, Cropland Data Layer Program. *Geocarto International 26*(5): 341–358. https://doi.org/10.1080/10106049.2011.562309 .

Breheny, P. and J. Huang. 2011. Coordinate descent algorithms for nonconvex penalized regression, with applications to biological feature selection. *Annals of Applied Statistics 5*(1): 232–253. https://doi.org/10.1214/10-AOAS388 .

Breiman, L. 2001. Random forests. *Machine Learning 45*(1): 5–32. https://doi.org/10.1023/A:1010933404324 .

Hastie, T.J. and D. Pregibon. 1992. Generalized linear models, In *Statistical models in S*, eds. Chambers, J.M. and T.J. Hastie, 195–247. Routledge.

Insolia, L., A. Kenney, F. Chiaromonte, and G. Felici. 2021. Simultaneous feature selection and outlier detection with optimality guarantees. *Biometrics* in-press. https://doi.org/10.1111/biom.13553 .

Karl, T. and W. Koss. 1984. *Regional and National Monthly, Seasonal, and Annual Temperature Weighted by Area, 1895-1983.* Historical climatology series. National Climatic Data Center.

Kuhn, M. 2009. Building predictive models in r using the caret package. *Journal of Statistical Software 28*(5): 1–26. https://doi.org/10.18637/jss.v028.i05 .

Naug, D. 2009. Nutritional stress due to habitat loss may explain recent honeybee colony collapses. *Biological Conservation 142*(10): 2369–2372. https://doi.org/10.1016/j.biocon.2009.04.007 .

PRISM Climate Group. 2022. Oregon State University. http://www.prism.oregonstate.edu (accessed in August, 2022).

R Core Team 2021. *R: A Language and Environment for Statistical Computing.* Vienna, Austria: R Foundation for Statistical Computing. https://www.R-project.org.

Redlands, C.E.S.R.I. 2021. ArcGIS Pro: Release 2.8.3. https://www.esri.com/en-us/arcgis/products/arcgis-pro/resources.

Riani, M., A.C. Atkinson, A. Corbellini, A. Farcomeni, and F. Laurini. 2022. Information criteria for outlier detection avoiding arbitrary significance levels. *Econometrics and Statistics* in-press. https://doi.org/10.1016/j.ecosta.2022.02.002 .

Rousseeuw, P.J. and K.V. Driessen. 1999. A fast algorithm for the minimum covariance determinant estimator. *Technometrics 41*(3): 212–223. https://doi.org/10.1080/00401706.1999.10485670 .

Simon, N., J. Friedman, T. Hastie, and R. Tibshirani. 2011. Regularization paths for cox's proportional hazards model via coordinate descent. *Journal of Statistical Software 39*(5): 1–13. https://doi.org/10.18637/jss.v039.i05 .

Todorov, V. and P. Filzmoser. 2009. An object-oriented framework for robust multivariate analysis. *Journal of Statistical Software 32*(3): 1–47. https://doi.org/10.18637/jss.v032.i03 .

United States Department of Agriculture, National Agricultural Statistics Service. 2022. Honey bee colonies. https://usda.library.cornell.edu/concern/publications/rn301137d (accessed in August, 2022).

Wright, M.N., T. Dankowski, and A. Ziegler. 2017. Unbiased split variable selection for random survival forests using maximally selected rank statistics. *Statistics in Medicine 36*(8): 1272–1284. https://doi.org/10.1002/sim.7212 .

Wright, M.N. and A. Ziegler. 2017. ranger: A fast implementation of random forests for high dimensional data in C++ and R. *Journal of Statistical Software 77*(1): 1–17. https://doi.org/10.18637/jss.v077.i01 .
